# Proteins of the cancer cell secretome induce the protumoral microenvironment of diffuse intrinsic pontine glioma

**DOI:** 10.1093/noajnl/vdaf132

**Published:** 2025-06-19

**Authors:** Merce Baulenas-Farres, Sonia Paco, Federica Marino, Jacqueline Mohr, Carla Panisello, Leire Balaguer-Lluna, Rosario Aschero, Maria Cuadrado-Vilanova, Claudia Resa-Pares, Eva Rodriguez, Jesus Marquez, Pablo Menéndez, Cristina Jou, Raul Benitez, Daniel Benitez-Ribas, Cinzia Lavarino, Jaume Mora, Angel M Carcaboso

**Affiliations:** Pediatric Cancer Program, Institut de Recerca Sant Joan de Deu (IRSJD), Barcelona 08950, Spain; SJD Pediatric Cancer Center Barcelona, Hospital Sant Joan de Deu, Barcelona, 08950, Spain; Pediatric Cancer Program, Institut de Recerca Sant Joan de Deu (IRSJD), Barcelona 08950, Spain; SJD Pediatric Cancer Center Barcelona, Hospital Sant Joan de Deu, Barcelona, 08950, Spain; Pediatric Cancer Program, Institut de Recerca Sant Joan de Deu (IRSJD), Barcelona 08950, Spain; SJD Pediatric Cancer Center Barcelona, Hospital Sant Joan de Deu, Barcelona, 08950, Spain; Pediatric Cancer Program, Institut de Recerca Sant Joan de Deu (IRSJD), Barcelona 08950, Spain; SJD Pediatric Cancer Center Barcelona, Hospital Sant Joan de Deu, Barcelona, 08950, Spain; Josep Carreras Leukemia Research Institute, Barcelona 08036, Spain; Pediatric Cancer Program, Institut de Recerca Sant Joan de Deu (IRSJD), Barcelona 08950, Spain; SJD Pediatric Cancer Center Barcelona, Hospital Sant Joan de Deu, Barcelona, 08950, Spain; Pediatric Cancer Program, Institut de Recerca Sant Joan de Deu (IRSJD), Barcelona 08950, Spain; SJD Pediatric Cancer Center Barcelona, Hospital Sant Joan de Deu, Barcelona, 08950, Spain; Pediatric Cancer Program, Institut de Recerca Sant Joan de Deu (IRSJD), Barcelona 08950, Spain; SJD Pediatric Cancer Center Barcelona, Hospital Sant Joan de Deu, Barcelona, 08950, Spain; Pediatric Cancer Program, Institut de Recerca Sant Joan de Deu (IRSJD), Barcelona 08950, Spain; SJD Pediatric Cancer Center Barcelona, Hospital Sant Joan de Deu, Barcelona, 08950, Spain; Pathology, Hospital Sant Joan de Deu, Barcelona 08950, Spain; Biobank, Hospital Sant Joan de Deu, Barcelona 08950, Spain; Institució Catalana de Recerca i Estudis Avançats (ICREA), Barcelona, Spain; Centro Investigación Biomédica en Red en Oncología (CIBER-ONC), ISCIII, Madrid, Spain; Josep Carreras Leukemia Research Institute, Barcelona 08036, Spain; Pediatric Cancer Program, Institut de Recerca Sant Joan de Deu (IRSJD), Barcelona 08950, Spain; SJD Pediatric Cancer Center Barcelona, Hospital Sant Joan de Deu, Barcelona, 08950, Spain; Biobank, Hospital Sant Joan de Deu, Barcelona 08950, Spain; Pathology, Hospital Sant Joan de Deu, Barcelona 08950, Spain; Health Technologies and Innovation Program, Institut de Recerca Sant Joan de Deu (IRSJD), Barcelona 08950, Spain; Automatic Control Department, Universitat Politecnica de Catalunya (UPC-BarcelonaTECH), Barcelona 08034, Spain; Immunology, Hospital Clinic de Barcelona, Barcelona 08036, Spain; Institut d’Investigacions Biomediques August Pi Sunyer, Barcelona 08036, Spain; Pediatric Cancer Program, Institut de Recerca Sant Joan de Deu (IRSJD), Barcelona 08950, Spain; SJD Pediatric Cancer Center Barcelona, Hospital Sant Joan de Deu, Barcelona, 08950, Spain; Pediatric Cancer Program, Institut de Recerca Sant Joan de Deu (IRSJD), Barcelona 08950, Spain; SJD Pediatric Cancer Center Barcelona, Hospital Sant Joan de Deu, Barcelona, 08950, Spain; Pediatric Cancer Program, Institut de Recerca Sant Joan de Deu (IRSJD), Barcelona 08950, Spain; SJD Pediatric Cancer Center Barcelona, Hospital Sant Joan de Deu, Barcelona, 08950, Spain

**Keywords:** diffuse intrinsic pontine glioma (DIPG), protein secretome, immune microenvironment, chitinase-3-like 1 (CHI3L1), osteopontin

## Abstract

**Background:**

The microenvironment of diffuse intrinsic pontine glioma (DIPG) is devoid of infiltrating lymphocytes and immune checkpoint molecules, with the exception of B7-H3. Here, we studied whether the cancer secretome is a determinant of such tumor phenotype.

**Methods:**

We quantified immune histologic markers in paraffin-embedded DIPG samples and healthy brainstem controls. We identified and quantified cytokines in frozen tissue samples, DIPG culture supernatants, cerebrospinal fluid (CSF) and plasma from patients and controls. We studied the phenotype of mesenchymal cells, brain microvascular endothelial cells and macrophages following their exposure to DIPG secretomes.

**Results:**

We found profuse infiltration of anti-inflammatory CD163^+^ microglia/macrophages in the brains of 23 DIPG patients, compared to low levels in 5 controls. In DIPG, B7-H3 was predominantly expressed in cells of mesenchymal origin (CD90^+^) transformed to pericytes (PDGFRβ^+^). In frozen samples from 14 patients with DIPG and 4 controls, we identified a common secretome pattern, with osteopontin and chitinase-3-like 1 (CHI3L1) overexpressed in DIPG. Such proteins were abundant in the culture supernatants of 7 DIPG models. Osteopontin and CHI3L1 concentrations in the CSF of 18 patients were significantly higher than in 18 controls (*P *< 0.0001). In vitro, DIPG supernatants and recombinant osteopontin or CHI3L1 induced phenotypic changes in (i) mesenchymal cells, which turned into B7-H3^+^ pericyte-like cells, (ii) endothelial cells, which organized complex tube networks and overexpressed the blood-brain barrier marker BCRP, even in subcutaneous xenografts, and (iii) macrophages, which were polarized towards the M2-like type.

**Conclusions:**

DIPG cells secrete proteins that create an immunosuppressed niche.

**Key Points**

- DIPG is rich in CD163^+^ microglia and B7-H3^+^ pericytes of mesenchymal origin.
- DIPG shows a common secretome, with overexpressed osteopontin and chitinase-3-like 1.
- The DIPG secretome induces protumoral changes in macrophages, mesenchymal and endothelial cells.

**Importance of the Study**

The microenvironment of diffuse intrinsic pontine gliomas (DIPG) is non-inflammatory, but it remains unclear whether it is actively immunosuppressive and how the final immune phenotype is acquired in this tumor. Here, we analyzed brainstem samples from patients and controls, to find that DIPG is rich in CD163^+^ (M2-like) microglia/macrophages and B7-H3^+^ pericytes of mesenchymal origin. We found a common secretome pattern of DIPG, with the proteins osteopontin and chitinase-3-like 1 significantly overexpressed in cell lines, tumors and patient cerebrospinal fluid. The DIPG secretome-induced protumoral changes in mesenchymal cells, which turned into B7-H3^+^ pericyte-like cells, endothelial cells, which organized complex tube networks and overexpressed the blood-brain barrier marker BCRP, and macrophages, which were polarized towards the M2-like type. In summary, the DIPG secretome actively promotes an immunosuppressive microenvironment, which should be taken into account for immunotherapeutic approaches.

Pediatric-type diffuse high grade gliomas (pHGG) are brain tumors characterized by glial, infiltrative and diffuse histology, high biological aggressiveness and dismal prognosis.^1^ Around 80% of pHGG belong to the subclass of diffuse midline gliomas (DMG), which are defined by a global loss of histone H3 K27 trimethylation (DMG, H3 K27-altered) due to either H3 K27M mutations in genes *HIST1H3B* and *H3F3A*, resulting in the substitution of lysine for methionine in the histone variants H3.1 and H3.3,^2,3^ or, more rarely, to aberrant expression of EZHIP, an H3 K27M onco-histone mimic.^4^ Around 80% of DMG are diagnosed as diffuse and expansive lesions of the brainstem, also known as diffuse intrinsic pontine gliomas (DIPG),^5^ which usually evolve to infiltrate the neighboring thalamus and cerebellum. These properties erase the possibility of surgical extirpation and contribute to only 10% of patients surviving longer than 2 years from diagnosis, rendering DIPG the leading cause of death of brain cancer in the pediatric age.

One of the factors likely contributing to the poor prognosis of diffuse gliomas is their immunosuppressive microenvironment.^6,7^ Adult-type diffuse gliomas show a “non-inflamed” tumor niche, with abundant CD163^+^ (M2-like) anti-inflammatory microglia^8^ and low-to-moderate number of CD3^+^ tumor-infiltrating lymphocytes (TILs).^9^ These tumors express suppressive immune checkpoint molecules such as programmed death ligand 1 (PD-L1). In adult-type gliomas, lymphocyte infiltration and PD-L1 are more abundant in tumors of higher grade,^10^ but anti-PD-L1 immunotherapy has not met the initial clinical expectations.^11^ The reason for the failure of this therapy could be related to glioma cells producing immunosuppressive cytokines such as transforming growth factor β (TGF-β), which inhibit immune cells.^12^

The phenotype of the immune microenvironment of DMG is also non-inflammatory, although others define it as “not clearly immunosuppressive.”^6,7^ Comparative studies of DMG with adult gliomas and pHGG (other than DMG) found that DMG has fewer number of TILs,^7^ very low or absent expression of PD-L1,^6,7^ and fewer counts of microglia or tumor associated macrophages (TAMs).^6^ These findings led to the hypothesis that immune surveillance is low in DMG and thus immunotherapies that recruit immune cells to these tumors could be successful treatments.^6^ Because B7 homolog 3 protein (B7-H3) was the main immune checkpoint molecule detected in DMG tissue samples, it was capitalized as a strategy to effectively activate the immune system in clinical trials.^13–15^ The therapeutic benefit provided by the anti-B7-H3 trials remains under evaluation.

Whether there exists a common paracrine DMG cell-driven mechanism leading to the described “neither inflammatory nor suppressive” immune environment remains not totally elucidated. One of the reasons for such incomplete knowledge is the scarcity of tissue samples (tumors and non-tumor controls obtained from similar anatomic locations) available for analysis. In this work, we studied a sufficiently large number of frozen samples, including healthy brainstem controls and DIPG (biopsies and autopsies), to find soluble immunoregulatory proteins present in DIPG at a consistently higher level than in the controls. We addressed the presence of the selected factors in the supernatants of primary DIPG cells in culture and in patient cerebrospinal fluid (CSF). We studied whether culture media conditioned with the DIPG secretome proteins induced phenotypic changes in cells likely present in the brain tumor microenvironment, such as mesenchymal stem cells turning into pericytes, brain microvascular endothelial cells turning into more differentiated vascular structures with blood-brain barrier (BBB) properties, and human macrophages turning into the M2-like CD163^+^ type.

## Materials and Methods

Detailed methods are provided in the Supplementary Methods section.

### Cancer Models and Patient Samples

We obtained primary pHGG cultures (identifications in Table S1) from the repository at Hospital Sant Joan de Deu (HSJD, Barcelona, Spain). All of them were established from tumors located in the pons (i.e. DIPG) and were classified as DMG H3 K27-altered. In the text, we omit the institutional prefix HSJD of cell identification, for clarity purposes.

The institutional review board at HSJD approved the collection of patient samples (DIPG from biopsy or autopsy, CSF or serum), under protocol PIC-157-15 and informed consent M-1608-C, through the HSJD Biobank. Other samples obtained under consent were from the BioBank HGUA (PT13/0010/0044), integrated in the Spanish National Biobank Network.

Adipose tissue-derived human mesenchymal stem cells (hMSC-AT, C-12977) were from PromoCell (Heidelberg, Germany), the human brain microvascular endothelial cell line hCMEC/D3 (SCC066) was from Sigma-Aldrich (Saint Louis, MO, USA), human astrocytes (K1884) were from Gibco (Grand Island, NY, USA), human microglia HMC3 (CRL-3304) was from ATCC (Manassas, VA, USA) and the cancer cell lines LAN-1 (neuroblastoma), A4573 (Ewing sarcoma) and RH (rhabdomyosarcoma) were from the HSJD repository.

Animal experiments followed the Institutional and European guidelines (EU Directive 2010/63/EU) and were approved by the local animal care and use committee from the University of Barcelona, Spain (protocol 134/18). We used 7 human cancer xenografts established in mice in our previous work^16–18^ (Table S2). Two models were established from patients with pHGG and 5 were from patients with extracranial cancers such as Ewing sarcoma, neuroblastoma, Wilms tumor and rhabdomyosarcoma (Table S2). Engraftment sites were orthotopic (intracranial) and subcutaneous (s.c.) for pHGG, and only s.c. for the extracranial cancer models. For this work, we used formalin-fixed paraffin-embedded (FFPE) tissue slides of the mentioned xenografts.

### Immunostaining of Patient Samples

We processed 4-μm FFPE tissue sections to characterize immune cell populations and tumor markers.

### Real-Time Quantitative Polymerase Chain Reaction (RT-qPCR)

We quantified mRNA expression of immune checkpoints and cytokines from brain and tumor tissue samples and primary DIPG cell cultures. Taqman Gene Expression Assays are detailed in Table S3. In brain endothelial cells, we analyzed gene expression of angiogenesis markers using a TaqMan™ Human Angiogenesis Array (4414071, Thermo Fisher Scientific, Waltham, MA, USA). For microglial cells and peripheral blood mononuclear cells (PBMC), we addressed macrophage polarization using the GeneQuery™ Human Macrophage Polarization Markers qPCR Array Kit (GK120, ScienCell Research Laboratories, Carlsbad, CA, USA).

### Induction of Immune Checkpoints Expression in DIPG

To evaluate whether the immune checkpoint machinery could be activated by a pro-inflammatory niche, we exposed DIPG cells and human astrocytes in culture (5 × 10^5^ cells per well, in 6-well plates) to 20 ng/mL interferon-γ (IFN-γ, 300-02, Peprotech, Cranbury, NJ, USA) for 48 h. We then used RT-qPCR to quantify *CD274* (PD-L1), *CD273* (PD-L2) and *CD276* (B7-H3) mRNA expression of treated and untreated cells (Table S3).

### Cytokine Expression in Patient Tissue Samples, Cell Culture Supernatants and Patient CSF and Serum

We assessed the presence of 105 human cytokines in frozen pHGG samples, brainstem control samples (obtained from pediatric patients without cancer and without CNS inflammation), and DIPG cell culture supernatants, using a proteome profiler array (Human XL Cytokine Array Kit, ARY022B, R&D Systems, Minneapolis, MN, USA). We compared the cytokine expression of control brainstem tissue and tumor samples and we identified the significantly overexpressed cytokines in tumor lysates using a parametric multiple t test.

To quantify selected proteins in tissue samples, DIPG cell supernatants, CSF and serum, we used ELISA kits for osteopontin and chitinase-3-like 1 (CHI3L1), or immunoblotting.

### Tumor Secretome-Induced Pericyte-Like Differentiation of Human Mesenchymal Stem Cells (hMSC)

To address whether the DIPG secretome-induced phenotypic changes of hMSC towards B7-H3-expressing pericytes frequently found in the aberrant blood vessels of DIPG, we incubated hMSC-AT with DIPG-conditioned culture medium supernatants for 7 days. We studied changes in the expression of B7-H3, NG2 and PDGFRβ by immunoblotting.

### Tumor Secretome-Induced Changes in Brain Endothelial Cells

First, we performed a microtubule formation assay of hCMEC/D3 cells exposed to DIPG supernatants and quantified the complexity of the vascular structures formed in vitro. Second, we studied phenotype changes in co-cultured mesenchymal cells and brain endothelial cells in the presence of DIPG secretomes. We also performed a microtubule formation assay of hCMEC/D3 cells co-cultured with hMSC-AT cells exposed to conditioned media.

### Tumor Secretome-Induced Changes in the Macrophage Phenotype

To address whether the DIPG secretome-induced polarization of the tumor microglia towards the M2-like phenotype, we exposed macrophage cultures to cell culture-conditioned medium. We isolated PBMC from blood buffy coats to perform the experiments. We expressed the results as the ratio of CD163^+^ cells (M2-like) to CD80^+^cells (M1-like). We also addresed the migration and invasion of macrophages in transwell assays.

### Statistical Analysis

We used GraphPad Prism 10 software for the statistical analysis (GraphPad, La Jolla, CA, USA). For the comparison of cytokine expression in tumors and controls of the proteome array we used the parametric t test corrected for multiple comparisons with the Holm-Sidak method. To compare numerical data between 2 different groups we used the Mann–Whitney test, and for more than 2 groups we used the Kruskal–Wallis test, both non-paired, non-parametric. For the comparison of more than 2 groups that follow a normal distribution and have similar SD, non-paired, we applied the ANOVA test. For all analyses, we considered P < 0.05 was a statistically significant difference.

## Results

### Comparative Infiltration of TILs and TAMs in DIPG and Non-Tumor Brainstem Samples

We obtained 25 FFPE tumor samples from 23 patients with pHGG, all DIPG, and 5 FFPE brainstem samples from 5 patients who died of causes other than brain cancer (Table S4). Tumor samples corresponded to 7 biopsies at initial diagnosis and 18 necropsies (2 patients had both biopsy and necropsy samples included in the analysis; Table S4). Among the 23 patients with DIPG, 20 had tumors classified as DMG H3 K27-altered. Fifteen of the 23 cases had molecular profiling of H3 mutations performed, being 12 of them H3.3 K27M-mutant, 2 H3.1 K27M-mutant and 1 wild type. Among the 8 DIPG samples without molecular studies, we found immunohistochemistry features suggestive of K27M histone mutations in 6 of them and 2 were radiologically classified as DIPG but the K27M status was not assessed (Table S4).

**Figure 1.**
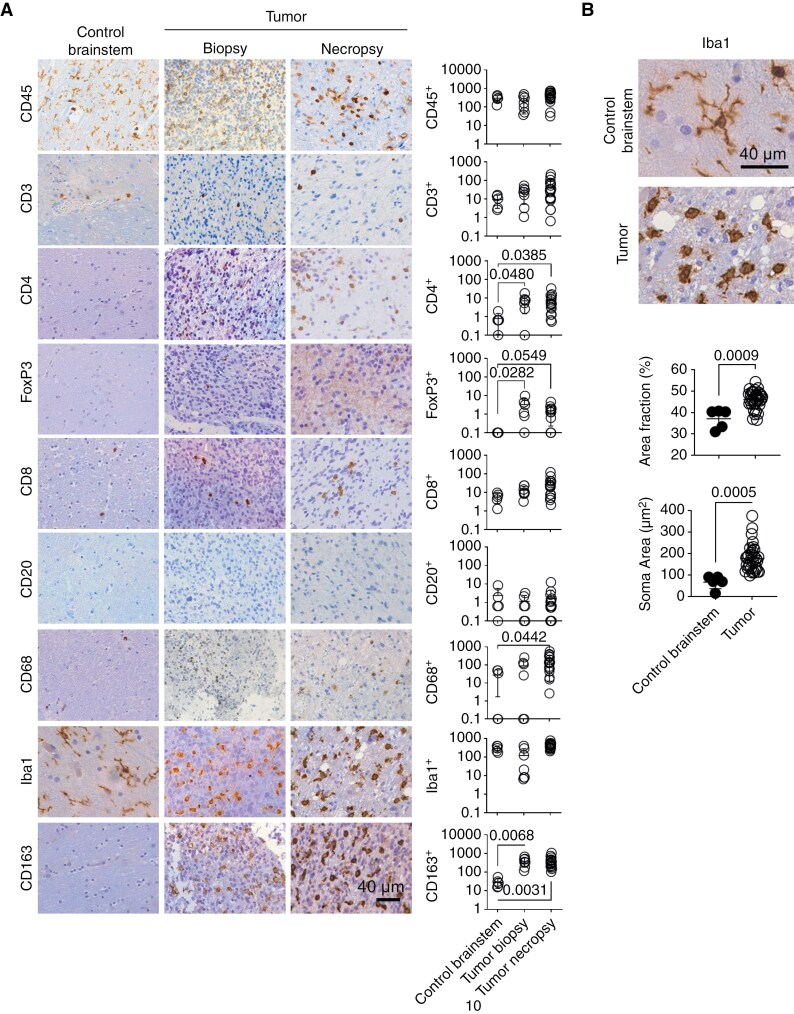
Quantification of TILs and TAMs in DIPG and control brainstem. (A) Representative images and quantification of CD45, CD3, CD4, FoxP3, CD8 CD20, CD68, Iba1 and CD163 stainings in control brainstem (n = 5), DIPG biopsies (n = 7) and DIPG necropsies (n = 17). Units of the y axis are cells/mm^2^. Statistics: Kruskal–Wallis test with Dunn’s multiple comparisons. (B) Representative images and morphology analysis of Iba1 in control brainstem (n = 5) and DIPG samples (n = 34 tumor samples, obtained from 19 patients). Dots in the graphics (panels A and B) are data from individual patient samples (1 dot per sample). Samples in which the count was 0 are represented as 0.1 in the logarithmic scale. Statistics: t test.

For analysis, we grouped separately tumor biopsies and necropsies. We immunostained 9 consecutive FFPE slides of each patient sample, 1 for each marker. Staining of immune cells with the marker CD45 was profuse in all samples, including nontumor-controls (Figure 1A). The density of the T lymphocyte population (CD3^+^ cells) was low in the control brainstem samples and slightly higher in DIPG biopsies and necropsies (Figure 1A). T helper (CD4^+^) and Treg (FoxP3^+^) cells were very low (1–2 cells/mm^2^ and undetectable, respectively) in control non-tumor samples, while they were significantly more abundant in cancer samples (Figure 1A). Cytotoxic T cells (CD8^+^) counts were similar to CD3^+^ counts and not significantly different between tumor and controls (Figure 1A). B cells (CD20^+^) were very scarse in all samples (Figure 1A).

There were 2 patterns of CD45 staining, CD45^high^, corresponding to round lymphocytes, and CD45^low^, corresponding to microglia/macrophages. CD45^low^ cells were the most abundant and presented a more ramified shape in the control brainstem than in tumors (Figure S1). CD68^+^ macrophages infiltrated most samples and they were significantly more abundant in the tumor necropsies, compared to control brainstem samples (Figure 1A). Iba1^+^ microglia/macrophages were equally abundant in control brainstem, biopsies and necropsies (Figure 1A). The finding of CD163^+^ M2-like TAMs in DIPG was very significant. They were abundant in tumor samples (necropsies and biopsies), and rare in brainstem controls (Figure 1A). In tumors, Iba1^+^ microglia/macrophages were bushy and round, in contrast to the ramified morphology observed in healthy brainstem (Figure 1B). Iba1^+^ cells in tumors showed higher area fraction and soma area than in control brainstems (Figure 1B).

**Figure 2.**
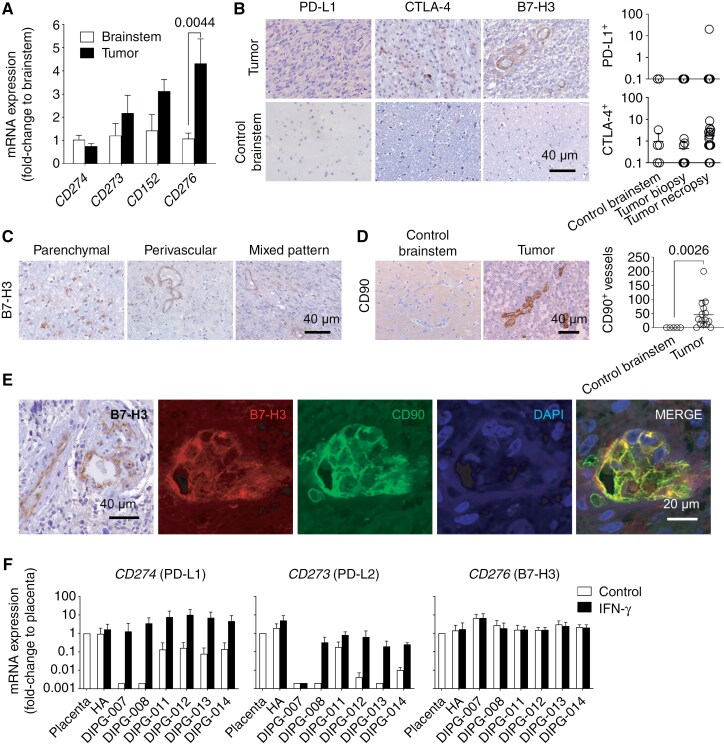
Expression of immune checkpoints in DIPG. (A) RT-qPCR analysis of the expression of genes *CD274, CD273, CD152* and *CD276* in tissue from control brainstem (n = 3) and tumor (n = 11) samples. Statistics: Mann–Whitney test. (B) Representative images and quantification of PD-L1, CTLA-4 and B7-H3 immunostaining in control brainstem (n = 5), DIPG biopsies (n = 7) and DIPG necropsies (n = 17). Samples in which the count was 0 are represented as 0.1 in the graphics. B7-H3 immunostaining was not quantified, due to the presence of 3 patterns of staining. Statistics: Kruskal–Wallis test with Dunn’s multiple comparisons. (C) Representative images of B7-H3 staining in DIPG for the parenchymal, perivascular and mixed patterns. (D) Representative immunohistochemistry and quantification of CD90^+^ vessels in control brainstem and DIPG. (E) Immunofluorescence of B7-H3 and CD90 in a DIPG sample with perivascular expression of B7-H3. (F) *CD274, CD273* and *CD276* mRNA expression in DIPG cell models incubated with normal pHGG medium (control) or pHGG medium with 20 ng/mL IFN-γ. Data are mean and SD of 3 independent experiments. Undetectable expression values are represented as 0.002.

Expression of immune checkpoints and escape mechanisms in DIPG. Upon analysis of gene expression in tissue from 4 control brainstems and 12 tumor samples (samples identified in Table S5), we found that gene *CD276* (B7-H3) was significantly overexpressed in tumors, compared to brainstem controls, while *CD274* (PD-L1), *CD273* (PD-L2) and *CD152* (CTLA-4) were not (Figure 2A). In paraffin samples (Table S4; all DIPG), we found PD-L1^+^ cells only in 1 tumor of 16 analyzed (of note, the positive tumor was the only one without H3 mutation in the series) and scarse CTLA-4^+^ cells in 14 of 18 tumors (Figure 2B). B7-H3 staining was positive in all (13/13) analyzed tumor samples (Figure 2B). None of the control brainstem samples expressed PD-L1 or B7-H3, while 60% of them (3 of 5) expressed CTLA-4 in a minority of cells (Figure 2B). We found 3 patterns of B7-H3 expression in tumors. In one-third of the samples (4 of 13), B7-H3 was exclusively expressed by vascular structures (perivascular and endothelial cells) (Figure 2C). In another third, B7-H3 was expressed only by cancer cells (Figure 2C). In the remaining samples (5 of 13), B7-H3 had an intermediate expression pattern, both in cancer cells and vascular structures (Figure 2C). The vasculature was positive for the mesenchymal marker CD90 in DIPG samples, while no control brainstem was positive for CD90 (Figure 2D). CD90^+^ staining colocalized with B7-H3 (Figure 2E).

Following the addition of IFN-γ to primary DIPG cells in culture, the expression of *CD274* (PD-L1) increased in all cases (P = 0.0312; t test with Wilcoxon matched-pairs test; Figure 2F). *CD273* (PD-L2) expression also increased in most of the cell models (P = 0.0625). In contrast, *CD276* (B7-H3) did not change (Figure 2F).

Last, we analyzed mRNA levels of additional immunoregulatory mechanisms of brain cancers.^19–24^ Regarding the kynurenine pathway, the expression of the gene encoding for the enzyme tryptophan-2,3-dioxygenase 1 (*TDO*) was similar in control and tumor samples, while we did not detect indoleamine 2,3-dioxygenase 1 (*IDO1*) or indoleamine 2,3-dioxygenase 2 (*IDO2*) genes (Figure S2A). Regarding cyclooxygenase-2 prostaglandin E2 pathway-related genes (prostaglandin E synthase and prostaglandin E synthase 2; *PTGES* and *PTGES2*), we did not detect changes in DIPG patients (Figure S2B). HLA class I and II molecules were slightly increased in tumors, although only significantly for the gene *HLA-DRA* (Figure S2C). The expression of the TGF-β isoform *TFGB2* was significantly increased in tumors (Figure S2D). We observed slight increases in tumor samples for heat shock proteins (HSP), only significant for *HSPA1A* (Figure S2E). The expression of *STAT3*, high mobility group box 1 (*HMGB1*) and hypoxanthine guanine phosphoribosyltransferase 1 (*HPRT1*) was not significantly different in tumors and controls, while calreticulin (*CALR*) was downregulated in tumors (Figure S2F).

### DIPG Secretome

**Figure 3.**
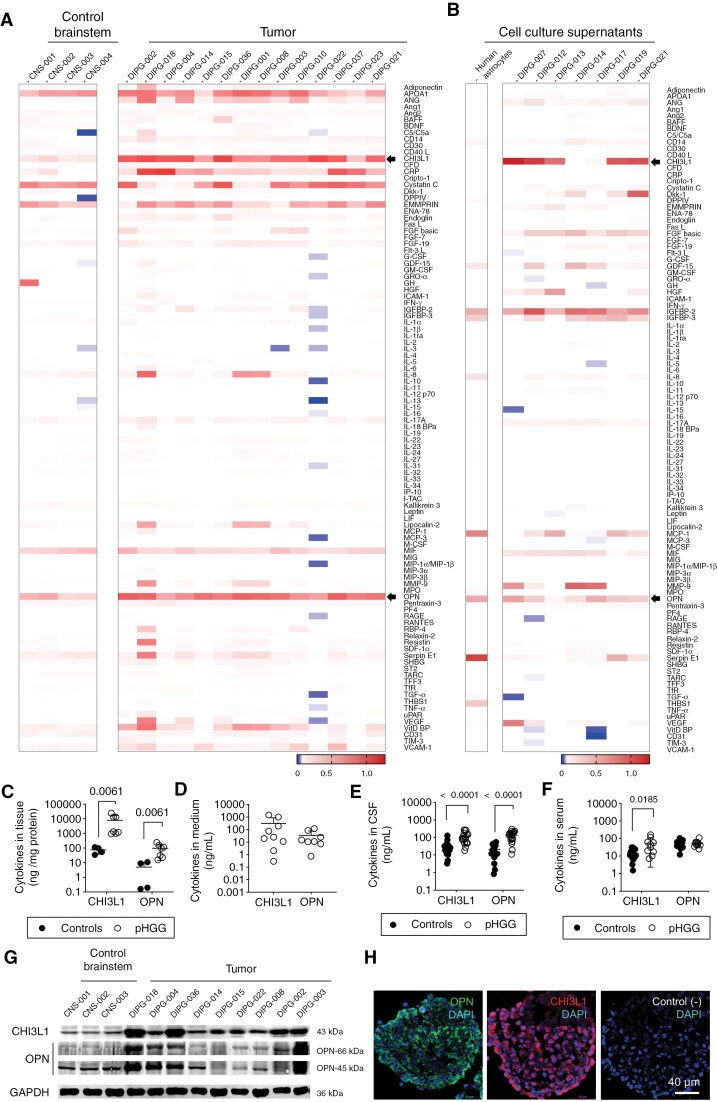
Analysis of the secretome of pHGG. (A) Heatmap representation of the cytokine array of the tissue homogenates of control brainstem samples and DIPG samples. Arrows indicate proteins significantly enriched in DIPG, CHI3L1 and osteopontin (OPN). Statistics: t test with Holm-Šídák’s multiple comparisons. (B) Heatmap representation of the cytokine array of supernatants of human astrocytes and DIPG primary cells in culture. Arrows indicate CHI3L1 and osteopontin. (C) Quantification of CHI3L1 and osteopontin in frozen samples of brainstem (controls; n = 4) or tumors (pHGG; n = 7). Statistics: Mann–Whitney test. (D) Quantification of CHI3L1 and osteopontin in pHGG supernatants (n = 8–9). (E) Quantification of CHI3L1 and osteopontin in patient CSF samples (n = 18 each). Mann–Whitney test. (F) Quantification of CHI3L1 and osteopontin in serum samples of controls (n = 15) and patients (n = 10). Mann–Whitney test. (G) Immunoblotting of CHI3L1 and osteopontin in brainstem controls and DIPG tissue samples. (H) Immunofluorescence of CHI3L1 and osteopontin in DIPG tumorspheres (DIPG-007 line). We show a negative control (sample incubated without primary antibody) for comparison.

We analyzed homogenized brainstem samples obtained from necropsies of 14 patients with DIPG and 4 patients without brain tumors (samples identified in Table S6). Representative images of the array membranes tests for 105 cytokines are in Figure S3A. We found that only 2 cytokines, CHI3L1 and osteopontin, were significantly enriched in DIPG, compared to control brainstem samples (Figure 3A). We did not detect expression of cytokines such as IL-1, IL-2, IL-4, IL-6, IL-7, IL-9, IL-10 or IL-13, proangiogenic factors such as VEGF, or immunoactivators such as TNF-α or IFN-γ (Figure 3A).

To test whether the identified proteins were secreted by DIPG cells in culture, we analyzed the cytokine profile of the supernatants of 7 primary DIPG models and the human astrocyte cell line for comparison. We detected CHI3L1 and osteopontin in the supernatants of 5 of 7 DIPG models (Figure 3B). Osteopontin was also secreted by human astrocytes, while CHI3L1 was exclusively secreted by DIPG cells (Figure 3B). Representative images of the test are in Figure S3A.

Next, we quantified (ELISA) the top-2 secreted cytokines, CHI3L1 and osteopontin, in samples (cell culture supernatants and homogenized tissues identified in Table S7). In frozen tissue samples, median CHI3L1 and osteopontin concentrations were around 100 and 10 times higher, respectively, in tumor samples than in controls (Figure 3C). CHI3L1 and osteopontin concentrations in tissue samples correlated well with the signals obtained in the protein arrays of the same samples (Figure S3B). In DIPG cell supernatants, CHI3L1 and osteopontin were detectable by ELISA in all the cell models (Figure 3D). Such levels correlated well with the signals obtained in the protein arrays in supernatants (Figure S3C). In agreement with the protein expression data, mRNA levels of the the genes *CHI3L1* and *SPP1* were also higher in tumor samples than in controls (Figure S3D).

Then, we analyzed the CSF of 18 pHGG patients (10 with confirmed DMG H3 K27-alterations, 1 with H3-wildtype and IDH-wildtype pHGG, and 7 cases with radiological diagnosis of DIPG) and 18 non-oncologic patients, free of inflammation (samples identified in Table S8). CHI3L1 concentration was significantly higher in pHGG (92 ng/mL; range 18–267 ng/mL) than in controls (20 ng/mL; 3–130 ng/mL) (Figure 3E). Osteopontin concentration was around 10 times higher in the tumor group (140 ng/mL; 11–299 ng/mL) compared to the controls (13 ng/mL; 1–55 ng/mL) (Figure 3E). In serum samples, the concentration of CHI3L1 was significantly higher in patients with pHGG (39 ng/mL; 7–165 ng/mL; n = 10) than in controls (12 ng/mL; 2–32 ng/mL; n = 9) (Figure 3F). Osteopontin levels in serum were similar in patients with pHGG (51 ng/mL; 25–111 ng/mL, n = 10) and controls (49 ng/mL; 12–106 ng/mL; n = 15) (Figure 3F).

Using immunoblotting, we detected higher levels of CHI3L1 and osteopontin (in its full-length –66 kDa- and cleaved -45 kDa- forms) in most of the frozen DIPG samples (identified in Table S9), compared to brainstem controls (Figure 3G). We detected the expression of these proteins in the cytoplasm of DIPG cells in culture (Figure 3H and S3E). In frozen patient samples, CHI3L1 and osteopontin immunostainings were significantly more intense in tumor samples than in control brainstem, and they were associated to SOX2^+^ cancer cells (Figure S3F).

### Secretome-Mediated Differentiation of Mesenchymal Stem Cells to B7-H3-Expressing Pericytes

**Figure 4.**
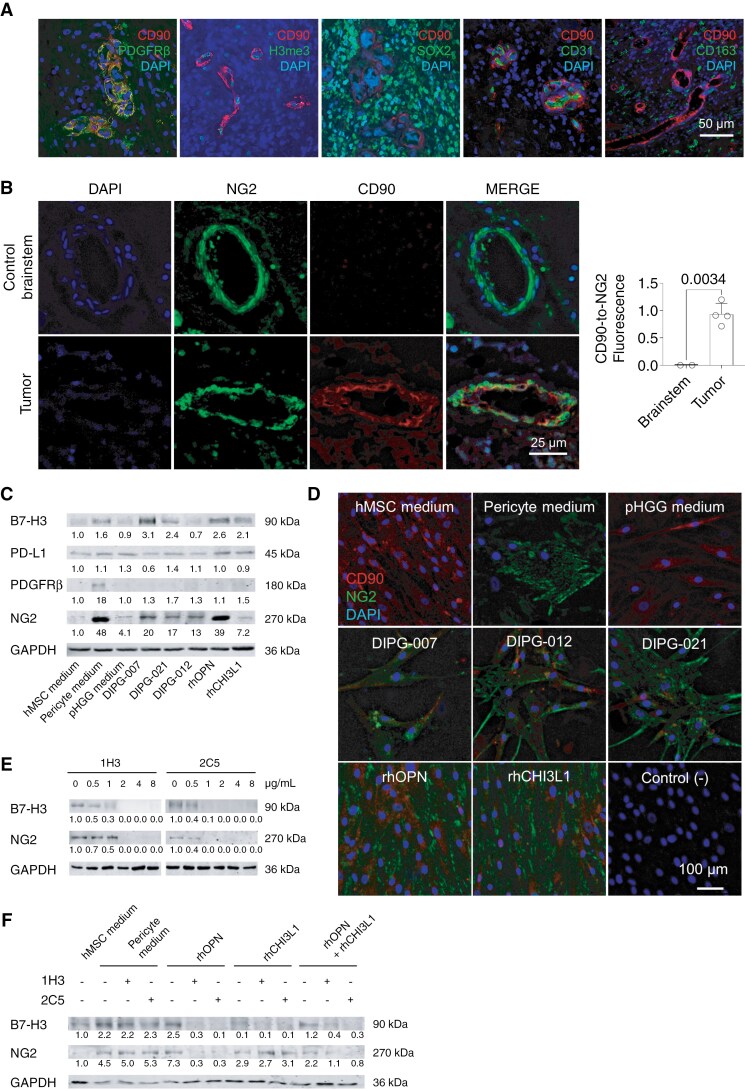
Pericyte differentiation in DIPG. (A) Representative confocal immunofluorescence of perivascular cells of mesenchymal origin (CD90^+^) in DIPG tissue, for markers CD90/PDGFRβ, CD90/tri-methyl-histone H3 (H3me3), CD90/SOX2, CD90/CD31, and CD90/CD163. (B) Representative immunofluorescence images and quantification of NG2 and CD90 in control brainstem (n = 2) and tumor tissue (n = 4) samples. Statistics: *t* test. (C) Immunoblotting of B7-H3, PD-L1, PDGFRβ and NG2 in hMSC-AT exposed for 7 days to fresh mesenchymal culture medium, pericyte medium, DIPG-conditioned media (3 cell lines), or recombinant osteopontin or CHI3L1 (rhOPN and rhCHI3L1). This experiment was repeated 3 times, with similar results. (D) Immunofluorescence of CD90 and NG2 in hMSC-AT cells incubated with conditioned media for 7 days. We show a negative control (tissue slides incubated without primary antibody) for comparison. (E) Immunoblotting of B7-H3 and NG2 in hMSC-AT cells exposed for 7 days to DIPG-conditioned media in the presence of osteopontin-neutralizing antibodies 1H3 and 2C5 at concentrations 0 to 8 µg/mL. (F) Immunoblotting of B7-H3 and NG2 in hMSC-AT exposed for 7 days to fresh mesenchymal culture medium, pericyte medium, rhOPN and rhCHI3L1, including their combination, in the presence and absence of osteopontin-neutralizing antibodies (2 µg/mL). (C,E,F) The numeric values under the bands indicate the fold change in protein levels relative to the control condition, which was set as 1.0. All protein levels were normalized to GAPDH.

In human DIPG samples, we observed that perivascular CD90^+^ B7-H3^+^ cells expressed the pericyte marker PDGFRβ (Figure 4A). As opposite to cancer cells, CD90^+^ cells were positive for the tri-methyl-histone H3 and negative for the tumor cell marker SOX2. Most CD90^+^ cells were also negative for the endothelial cell marker CD31 and all were negative for the TAM marker CD163 (Figure 4A). In contrast to tumor samples, control brainstem samples did not express CD90 in perivascular cells (Figure 4B; samples identified in Table S10). These results suggest that tumors in the brainstem, but not normal brainstems, contain cells of mesenchymal origin transformed to pericytes. To address this, we studied whether the DIPG secretome-induced a pericyte-like phenotype in CD90^+^ cells of the mesenchymal lineage in vitro. We found that the supernatant of 3 different DIPG cells, and recombinant osteopontin, induced the marker NG2 in hMSC-AT cells (Figure 4C,D). NG2 is associated to early-stage differentiated pericytes in the neovascularization process.^25^ The marker of late-stage pericyte differentiation, PDGFRβ, appeared only following the incubation in pericyte medium (Figure 4C). B7-H3 expression increased in mesenchymal cells exposed to 2 of the 3 DIPG supernatants, and to recombinant proteins osteopontin and CHI3L1, while PD-L1 expression did not change in any of the experimental conditions (Figure 4C). The supernatants of human astrocytes, LAN-1 or A4573 cells did not induce changes in B7-H3, PD-L1, NG2 and PDGFRβ expression in hMSC-AT cells (Figure S4A,B). Osteopontin-neutralizing antibodies at concentrations above 1 µg/mL inhibited the expression of NG2 and B7-H3 in hMSC-AT cells exposed to the secretome of DIPG (Figure 4E) or to recombinant osteopontin (Figure 4F). Overall, our experiments show that the secretome of DIPG cells, but likely not other cancer cells, induce the differentiation of mesenchymal cells into pericyte-like cells expressing B7-H3, and osteopontin is an important factor in this process.

### Promotion of Angiogenesis by the DIPG Secretome

**Figure 5.**
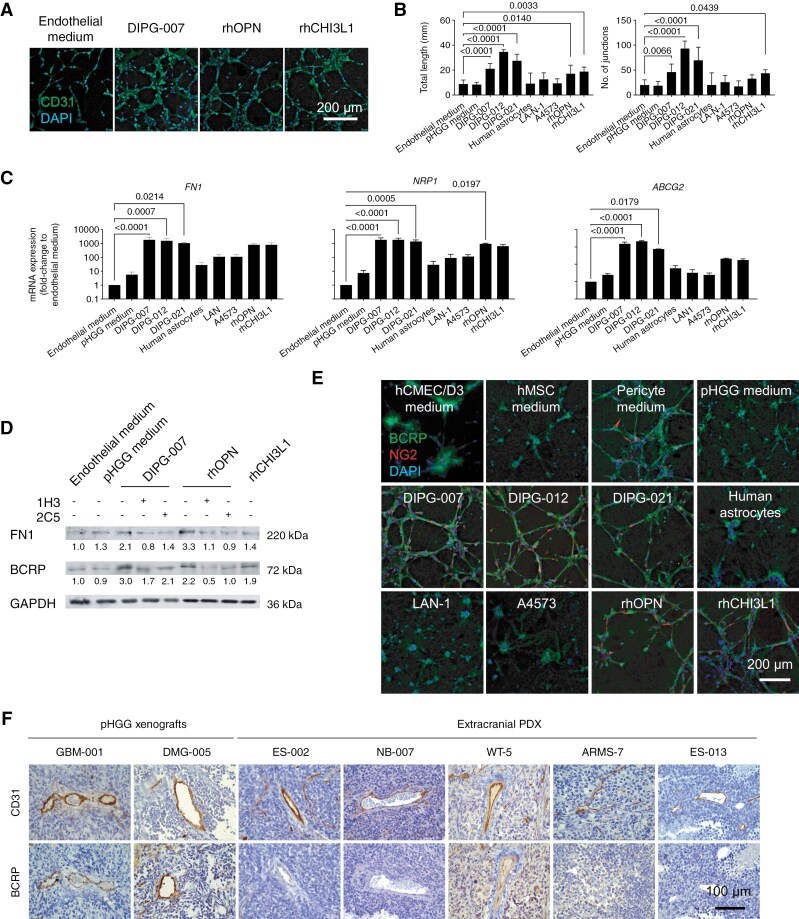
Effect of the DIPG secretome promoting angiogenesis and the BBB marker BCRP. (A) Representative images of the tube formation assay of hCMEC/D3 cells exposed to fresh culture medium, DIPG-conditioned medium (DIPG-007), and recombinant osteopontin and CHI3L1 (rhOPN and rhCHI3L1) for 18 h. Images show the immunofluorescence of CD31. (B) Quantification of the total tubule length and number of junctions of the endothelial cell nets. Statistics: 1-way ANOVA with Dunnett’s multiple comparisons. (C) RT-qPCR analysis of genes *FN1*, *NRP1* and *ABCG2*, relative to *GUSB*, in endothelial cells exposed to the conditions of the array. Values are means and SD of 3 independent experiments. Statistics: 1-way ANOVA with Dunnett’s multiple comparisons. (D) Immunoblotting of FN1 and BCRP in hCMEC/D3 cells exposed for 48 h to DIPG-conditioned medium (DIPG-007) or rhOPN, in the presence or absence of osteopontin-neutralizing antibodies 1H3 and 2C5 at 2 µg/mL, compared to control conditions and rhCHI3L1. The numeric values under the bands indicate the fold change in protein levels relative to the control condition, which was set as 1.0. All protein levels were normalized to GAPDH. The experiment was repeated 3 times. (E) Immunofluorescence of BCRP and NG2 in co-cultures of hMSC-AT and hCMEC/D3 cells exposed to fresh culture media, contitioned media (DIPG lines, human astrocytes, LAN-1 and A4573), rhOPN or rhCHI3L1 for 18 h. (F) Immunostaining of CD31 and BCRP in s.c. xenografts of pHGG (DMG-005 and GBM-001), and s.c. PDX of Ewing sarcoma (ES-002, ES-013), neuroblastoma (NB-007), Wilms tumor (WT-5) and rhabdomyosarcoma (ARMS-7).

DIPG supernatants and recombinant osteopontin or CHI3L1 induced tube networks of high complexity in hCMEC/D3 endothelial cells (Figure 5A and S5A). The network was less complex when the cells were cultured in their own endothelial medium, or when they were exposed to the supernatant of other cancer cell lines or human astrocytes (Figure S5A). The total tubule length and number of junctions was higher in the endothelial cells exposed to the DIPG-conditioned media (Figure 5B).

In hCMEC/D3 cells, the expression of genes related to the promotion of angiogenesis, such as fibronectin (*FN1*), neuropilin (*NRP1*), collagen type IV alpha (*COL4A*), forkhead box C2 (*FOXC2*) and granulin (*GRN*) increased 100–1000 fold following exposure to DIPG supernatants, recombinant osteopontin and CHI3L1 (Figure S5B). In contrast, supernatants of human astrocytes and cancer cell lines LAN-1 and A4573 induced discretely increased levels of around 10-fold (Figure S5C). We confirmed by RT-qPCR the array results for genes *FN1* and *NRP1* (Figure 5C). Remarkably, mRNA expression of the BBB marker *ABCG2* (BCRP) increased abruptly, around 100 times, in hCMEC/D3 cells exposed to DIPG supernatants, osteopontin and CHI3L1, while changes were modest following exposure to human astrocytes, LAN-1 or A4573 cell lines (Figure 5C). The expression of proteins fibronectin and BCRP increased in hCMEC/D3 cells exposed to DIPG-007 supernatant or recombinat osteopontin, compared to the basal condition. Neutralization of osteopontin inhibited such effect (Figure 5D).

Co-culturing endothelial and mesenchymal cells in the presence of the DIPG, osteopontin and CHI3L1-conditioned media resulted in complex networks of BCRP-expressing endothelial cells, surrounded by NG2-expressing pericyte-like mesenchymal cells, mimicking the basic structure of blood vessels (Figure 5E). Such networks did not form when the co-culture was exposed to supernatants of human astrocytes, LAN-1 or A4573 (Figure 5E). To evaluate whether BCRP expression would be reproduced in mouse xenografts (i.e. in endothelial cells recruited by the human tumor from the mouse host), we analyzed xenografts engrafted s.c. in mice. Unexpectedly, BCRP staining was positive in CD31^+^ blood vessels in s.c. pHGG xenografts (Figure 5F and S6A), while it was negative in all the PDX models derived from extra-CNS cancers, suggesting that pHGG cells, but not other cancers, favor the expression of BBB markers in the vasculature of the microenvironment (Figure 5F). We verified that the endothelial cells of pHGG models engrafted in intracranial location were also BCRP^+^ (Figure S6A). In all cases, we confirmed that the intratumoral endothelial structures were not of human origin, because they were negative for a human-specific marker (Figure S6B).

### Modulation of the Polarization and Migration Properties of Macrophages by the DIPG Secretome

**Figure 6.**
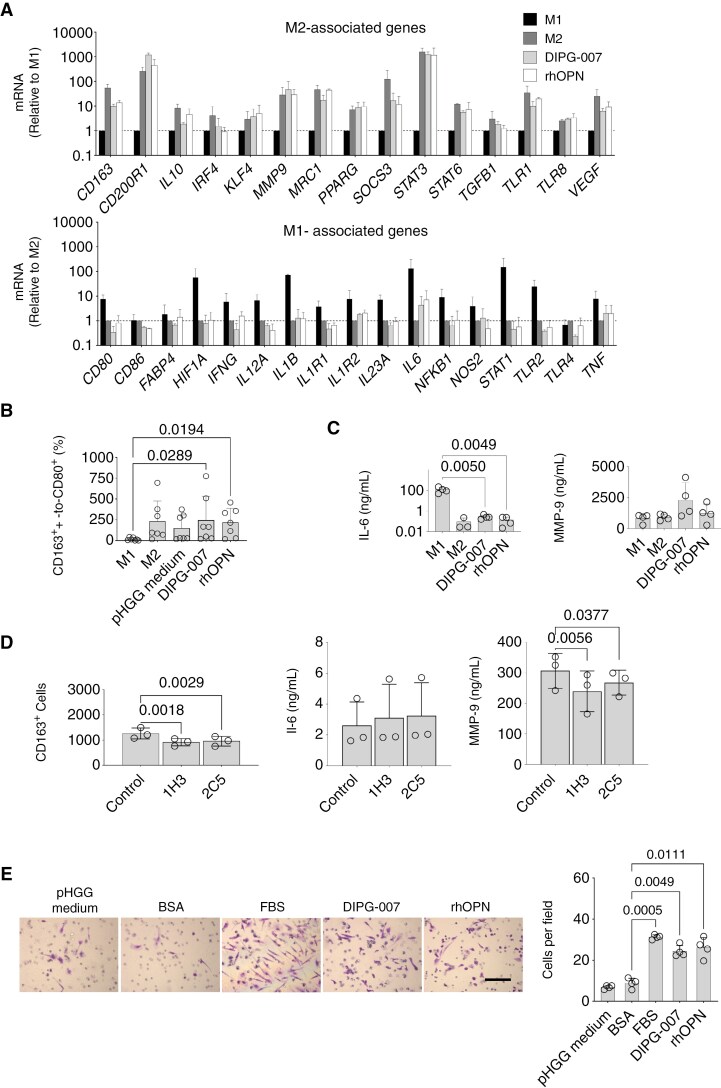
Effect of the DIPG secretome on the polarization and migration properties of PBMC-derived macrophages. (A) Expression of genes included in the GeneQuery™ Human Macrophage Polarization Markers qPCR Array Kit, in PBMC-derived macrophages exposed to polarizing cytokines cocktails (M1 and M2), DIPG-conditioned medium (DIPG-007), or recombinant osteopontin (rhOPN) for 24 h. Values of M1 and M2-associated genes are normalized to those obtained for macrophages exposed to M2 or M1-polarizing cocktails, respectively. Bars are means and SD of 2 independent experiments. (B) M2-like-to-M1-like (CD163^+^-to-CD80^+^) polarization ratios in the experimental groups of macrophages exposed to polarizing cytokines cocktails (M1 and M2), fresh pHGG medium, DIPG-conditioned medium (DIPG-007), or recombinant osteopontin (rhOPN) for 24 h. Bars and error bars represent means and SD obtained from 7 independent experiments (scattered dots), i.e. from 7 different donors. Statistics: Kruskal–Wallis test with Dunn’s multiple comparisons. (C) Concentration of IL-6 and MMP-9 in the supernatants of macrophages exposed to M1 and M2 cocktails, DIPG-conditioned medium (DIPG-007), or rhOPN for 24 h. Results from 4 experiments are shown. Statistics: 1-way ANOVA with Tukey’s multiple comparisons. (D) CD163^+^ count and concentration of IL-6 and MMP-9 in the supernatants of macrophage cultures after exposure to DIPG-conditioned medium (DIPG-007) in the absence (control) or presence of neutralizing antibodies 1H3 or 2C5, both at 2 μg/mL, for 24 h. Bars and error bars are means and SD from 3 different donors (1 dot per donor). Statistics: 1-way ANOVA with Dunnett’s multiple comparisons. (E) Representative images of macrophage invasion and migration in transwell assays. Cells were stained with crystal violet. Quantification of migration of macrophages in each condition. Bars and error bars represent means and SD obtained from 4 independent experiments (scattered dots). Statistics: 1-way ANOVA with Holm-Šídák’s multiple comparisons.

PBMC-derived macrophages exposed to M1 and M2 cytokine cocktails expressed M1 and M2-associated genes accordingly in the expression array (Figure 6A). When we incubated the macrophages with DIPG supernatant or recombinant osteopontin, genes associated to the M1 phenotype, such as *CD80*, *HIF1A*, and *IL6*, were downregulated, and genes associated to the M2 immunosuppressive phenotype, such as *CD163*, *MMP9*, and *STAT3*, were increased, at levels similar to those obtained for the M2 cocktail (Figure 6A).

In flow cytometry assays we found a significantly higher CD163^+^-to-CD80^+^ ratio in macrophages exposed to DIPG supernatant, compared to macrophages exposed to the M1 cocktail (Figure 6B). This phenomenon was characterized by a notable reduction in CD80^+^ cells and a moderate increase in CD163^+^ cells. Recombinant osteopontin induced effects similar to DIPG supernatant (Figure 6B). Concordantly with the results of gene expression, secreted protein IL-6 decreased significantly and MMP-9 increased in the macrophage culture supernatants exposed to the DIPG secretome (Figure 6C). Adding osteopontin-neutralizing antibodies to macrophages exposed to DIPG supernatant decreased significantly the number of CD163^+^ cells (Figure 6D), downregulated M2-associated genes and increased M1-related ones (Figure S7), and decreased significantly the secretion of the M2-associated protein MMP-9 (Figure 6D).

Finally, we observed that macrophages exposed to DIPG supernatant or recombinant osteopontin showed enhanced migration in transwell assays, compared to the control condition (Figure 6F).

## Discussion

This work demonstrates that DIPG cells secrete soluble factors that change the surrounding stroma cells to induce a favorable microenvironment for tumor expansion and growth. As a result of the paracrine signaling of DIPG, the tumor parenchyma shows very low infiltration of lymphocytes, abundance of M2-like microglia and frequent B7-H3^+^ pericytes of mesenchymal origin, reinforcing neovessels with enhanced BBB properties.

Our finding of sparse or absent TILs and B cells infiltrating the human tumor samples is in agreement with previously published data.^6,7^ However, the very significant increase of M2-like activated microglia (CD163^+^, Iba1^+^) in our DIPG cohort, compared to the low counts in location-matched samples from non-tumor patients, was not detected in a study comparing DIPG samples with apparently normal adjacent tissue obtained from surgical resection in the same patients.^6^ In such study, adjacent control sections from cancer patients had levels of CD163^+^ microglia similar to those detected in tumor areas. To explain the discrepancy, we suggest that tumor secretions could modify the microenvironment at distant sites, increasing CD163^+^ microglia in apparently normal samples devoid of cancer cells.

The fact that PD-L1 and CTLA-4 are not present in samples from patients with DIPG suggests that cancer cells did not receive previous stimulation from T cells.^6^ In the presence of an artificial inflammatory environment, we observed that DIPG cells expressed significant amounts of PD-L1 and CTLA-4, suggesting that the IFN-γ receptor pathway inducing checkpoint expression is conserved in DIPG cells, but it remains untriggered in the tumor.^26^ This has been previously observed for cultured cells of retinoblastoma^27^ and non-small cell lung cancer.^28^

Our finding of frequent B7-H3 expression in DIPG was first published by Zhou et al^14^ and justified clinical trials.^15^ B7-H3 inhibits the proliferation of CD8^+^ and CD4^+^ T cells^29^ and promotes the recruitment and M2-like polarization of the microglia.^30,31^ The predominant expression pattern of B7-H3 in human DIPG samples, in PDGFRβ^+^ pericytes of non-tumoral origin, was recently described for adult gliomas.^32^ In this sense, our work contributed to identify the DIPG secretome promoting the expression of B7-H3 in hMSCs, together with pericyte markers. The ability of brain cancers to recruit mesenchymal cells and transform them to pericytes was first reported in rat gliomas, in which bone marrow-derived mesenchymal cells migrated to perivascular location and expressed pericity markers following intratumoral injection.^33^ To our knowledge, ours is the first evidence that glioma cells induce B7-H3 in this type of stem cells transformed to pericytes.

The DIPG secretome causing increased expression of BBB proteins in endothelial cells was one of the most intriguing results of our work. In an important study, osteopontin produced by hematogenous macrophages was shown to restore the BBB by reducing protein extravasation after experimental photothrombotic brain infarction in mice.^34^ In previous work, we and others observed that gene and protein expression of *ABCG2* (BCRP) and other BBB markers such as P-glycoprotein were not significantly induced in co-cultures of human umbilical cord blood-derived endothelial cells and DIPG cell lines.^35^ In contrast, our present study found up to 250-fold increase in *ABCG2* and a very significant increase in more than 40 angiogenesis-related genes. The difference between both studies could be due to the use of different sources of endothelial cells, with possible involvement of the cerebral origin of the cells in our present work.^36^ Our analysis of s.c. human xenografts in mice confirmed the overexpression of BBB proteins in the in vivo setting, showing that only pHGG xenografts had vessels with BCRP. To our knowledge, this finding in xenografts is unique in the literature and demonstrates that pHGG cells induce their own reinforced vasculature, even in extracranial location. This does not mean, however, that the BBB was fully established in s.c. xenografts, because other factors such as the interstitial pressure or the microvascular endothelial network are not reproduced in the s.c. setting.^37^

The 2 proteins identified as common to most patients of our cohort might contribute to the malignancy of DIPG. CHI3L1 is overexpressed in cancer^38^ and relates to worse prognosis, macrophage recruitment and angiogenesis.^39^ Osteopontin is also associated with cancer, metastatic processes^40^ and immunosuppression, regulating M2-like polarization of microglia.^41^ Our experiments confirm their powerful capacity to induce neovascularization and, for the case of osteopontin, direct M2 macrophage polarization and migration. CHI3L1 and osteopontin levels in the patient CSF and serum are potential biomarkers for CNS diseases such as CNS lymphoma,^42^ histiocytosis^43^ and multiple sclerosis.^44–46^ In the case of traumatic brain injuries, resident cells in the CNS, likely the macrophages, secrete osteopontin to control the inflammation and repair the tissues, as demonstrated in models of ischemic stroke.^47,48^ Thus, we believe coherent that, by producing similar cytokines, DIPG maintains the immunoprivileged state of the CNS and create a favorable environment for tumor growth. Our study confirmed a significant overexpression of osteopontin and CHI3L1 in the CSF and serum of DIPG patients, but whether these proteins could be used as biomarkers for DIPG progression needs to be assessed in prospective work.

## Supplementary Material

vdaf132_suppl_Supplementary_Materials

vdaf132_suppl_Supplementary_Tables_S1-S10

vdaf132_suppl_Supplementary_Figures_S1-S7

## Data Availability

Data of this study are available from the corresponding author upon reasonable request.
