# Supplementary material for: Proteins of the cancer cell secretome induce the protumoral microenvironment of diffuse intrinsic pontine glioma
Source: Neurooncol Adv. 2025 Jun 19;7(1):vdaf132. doi: 10.1093/noajnl/vdaf132 (PMC12284641; doi:10.1093/noajnl/vdaf132)
Supplement: vdaf132_suppl_Supplementary_Materials [file vdaf132_suppl_supplementary_materials.docx]

**Supplementary methods**

**Cancer models and patient samples.** Primary pHGG cultures grow as tumorspheres in pHGG culture medium. The composition of this medium is neurobasal-A (reference 10888022) and DMEM/F-12 (11320074) (50:50 volume) supplemented with B-27 (12587010), HEPES buffer 10 mM (15630056), sodium pyruvate 1 mM (11360039), minimum essential medium (MEM) non-essential aminoacid solution 1x (11140035), L-alanyl-L-glutamine 2 mM (35050038), penicillin 100 units/mL, streptomycin 100 µg/mL and amphotericin B 0.25 µg/mL (15240062), all from Thermo Fisher Scientific (Waltham, MA, USA). Medium contains recombinant human growth factors EGF (AF-100-15) and FGF-basic (AF-100-18B) (20 ng/mL each), PDGF-AA (100-13A) and PDGF-BB (100-14B) (10 ng/mL each), from PeproTech (Cranbury, NJ, USA) and heparin 2 µg/mL (H3149-10KU, Sigma-Aldrich, Saint Louis, MO, USA).

**Immunostaining of patient samples.** For 4-μm paraffin-embedded tissue sections, we incubated the samples with peroxidase blocking solution (RE7101, Leica) for 30 min. Then, we washed the sections and we used primary antibodies for CD3 (PA0553, ready-to-use, Leica, Wetzlar, Germany), CD8 (ab17147, 1:50, Abcam, Cambridge, UK), CD4 (NCL-L-CD4-368, 1:200, Novocastra, Newcastle upon Tyne, UK), FoxP3 (ab20034, 1:400, Abcam), CD20 (MOB004, ready-to-use, Diagnostic Biosystems, Pleasanton, CA, USA), CD45 (PA0042, ready-to-use, Leica), CD68 (MOB167, ready-to-use, Diagnostic Biosystems), Iba1 (ab178846, 1:2000, Abcam), CD163 (NCL-L-CD163, 1:400, Novocastra), PD-L1 clone SP142 (M4420, 1:60, Spring Bioscience, Pleasanton, CA, USA), PD-L2 (#82723, ready-to-use, Cell Signalling, Danvers, MA, USA), CTLA-4 (anti-CD152 clone 14D3, 14-1529, 1:100, eBioscience, San Diego, CA, USA), B7-H3 (#14058, 1:200, Cell Signalling), CD31 (ab28364, 1:100, Abcam), breast cancer resistance protein (BCRP; OACD01214, Aviva Systems Biology, San Diego, CA, USA) and CD90 (ab181469, 1:200, Abcam). Secondary antibodies and diaminobenzidine solution for staining were those included in the Novolink system for the detection of mouse IgG, mouse IgM and rabbit IgG primary antibodies (RE7150, Leica).

For immunofluorescence, we pretreated 4-µm paraffin tissue sections with Sudan black 0.1% in 70% ethanol to remove tissue autofluorescence. After 20 min, we washed them with phosphate buffered saline (PBS) and blocked unspecific unions with 5% bovine serum albumin (BSA, A3294, Sigma-Aldrich) for 30 min. Then, we incubated the sections overnight at 4 ºC with primary antibodies diluted in blocking solution for osteopontin (ab8448, 1:400, Abcam), chitinase 3-like 1 (CHI3L1; ab77528, 1:1000, Abcam), SOX2 (ab171380, 1:400, Abcam), B7-H3 (#14058, 1:200, Cell Signalling), CD31 (ab32457, 1:1000, Abcam), CD90 (ab181469, 1:200, Abcam), CD163 (ab182422, 1:500, Abcam), PDGFRβ (#3169, 1:100, Cell Signalling) and tri-methyl-histone H3 (Lys27) (#9733, 1:1000; Cell Signalling). The next morning, we washed with PBS and added 4′,6-diamidino-2-phenylindole (DAPI; D1306, 1:5000, Thermo Fisher Scientific) and the secondary antibodies anti-rabbit-Alexa 594 (A11012, 1:100) and anti-mouse-Alexa 488 (A32723, 1:100) from Invitrogen. After 1 h in the dark, we washed the slides with PBS and mounted them with antifade mountant (P36961, Thermo Fisher Scientific). We obtained images using the Leica SP8 confocal microscope and acquired them with the Leica Application Suite software. To count cell populations, we took random photographs of five areas (0.3 mm^2^) of each sample with a 20x objective, and counted the number of positive cells manually. The mean of the five analyses was the count assigned to each sample.

**Morphology of the microglia in human tumor samples.** To determine the activation state of the microglia in the human samples, we analyzed the morphology of Iba1^+^ cells in paraffin sections. We used FracLac plugin for ImageJ (National Institutes of Health, Bethesda, MD, USA) and calculated the parameters of area fraction of Iba1^+^ cells in the selected field and soma area (i.e., the total number of pixels in the cell soma, multiplied by the pixel area of 0.013 µm^2^). We selected cells randomly, no overlapping with neighboring cells and with complete nucleus and branches.

**Real-time quantitative polymerase chain reaction (RT-qPCR).** We quantified mRNA expression of immune checkpoints and cytokines from brain and tumor tissue samples and primary DIPG cell cultures. We purified total RNA from cell models and tissue samples using TRIzol Reagent (15596018, Life Technologies, Carlsbad, CA, USA) following the manufacturer’s instructions. We determined the quantity and quality of RNA obtained with Nanodrop 1000 spectrophotometer (Thermo Fisher Scientific). A total of 1 μg of RNA was retrotranscribed with M-MLV reverse transcriptase system (Life Technologies) to obtain cDNA. We performed RT-qPCR according to the manufacturer’s protocol using Taqman Gene Expression Master Mix (4444557, Thermo Fisher Scientific) and Taqman Gene Expression Assays detailed in **Table S3**. The relative expression of the target genes was calculated using the ΔΔCt method with control *TBP*.

In brain endothelial cells, we performed RT-qPCR according to the manufacturer’s protocol using Power SYBR Green PCR Master Mix Gene Expression Master Mix (4367659, Thermo Fisher Scientific) and SYBR Gene primers for genes fibronectin 1 (*FN1*; Fw: ACAACACCGAGGTGACTGAGAC, Rv: ACAACACCGAGGTGACTGAGAC), neuropilin 1 (*NRP1*; Fw: AACAACGGCTCGGACTGGAAGA, Rv: GGTAGATCCTGATGAATCGCGTG) and ATP-binding cassette super-family G member 2 (*ABCG2*; Fw: AAAGCCACAGAGATCATAGAG, Rv: GATCTTCTTCTTCTTCTCACC). The relative expression of the target genes was calculated using the ΔΔCt method with control *GUSB* (Fw: CTGTCACCAAGAGCCAGTTCCT, Rv: GGTTGAAGTCCTTCACCAGCAG).

**Cytokine expression in patient tissue samples, cell culture supernatants and patient CSF and serum.** For the proteome profiler array, we homogenized tissues in lysis buffer (0.5% IGEPAL, 0.5% sodium deoxycholate, 0.1% sodium dodecyl sulfate, 50 mM Tris-HCl (pH 7.5), and 150 mM NaCl; all from Sigma) supplemented with protease inhibitor cocktail 0.4% (4693132001, Promega, Madison, WI, USA). We quantified protein concentration in homogenates or supernatants using the Bradford assay (B6916, Sigma-Aldrich). We added to the array membranes either patient tumor tissue lysates (200 µg protein in 1.5 mL of array buffer), or DIPG cell culture supernatant (300 µL). After overnight incubation at 4 °C, we added the biotinylated detection antibody cocktail and streptavidin-HRP. We acquired images using the iBrightCL1000 (Invitrogen) and quantified the intensity of the spots with QuickSpots HLIimage++ software (Ideal Eyes System, Bountiful, Utah, USA). Exposure time was 5 min for supernatant samples and 15 min for tissue samples. In each array membrane, we subtracted the signal of the negative control spots to each of the individual signals obtained from each protein spot. Then, we normalized the signal value of each of the spots to the mean signal value of the positive controls, which we considered 100% signal. We compared the cytokine expression between control brainstem tissue and tumor samples and we identified the significantly overexpressed cytokines in tumor lysates using a parametric multiple t test.

To quantify selected proteins in tissue samples, DIPG cell supernatants, CSF and serum, we used ELISA kits for osteopontin (DOST00) and chitinase 3-like 1 (abbreviated CHI3L1; DC3L10), both from R&D Systems. We read the plates using an Infinite 200 PRO TECAN microplate reader (Tecan Trading, Männedorf, Switzerland) set at 450 nm, with a wavelength correction set at 540 nm.

We addressed protein expression in tissue samples by immunoblotting of osteopontin and CHI3L1 using GAPDH as loading control. We blocked membranes for 1 h at room temperature (RT) with 5% BSA and incubated them overnight at 4 °C with the primary antibodies for osteopontin (ab8448, 1:1000, Cell Signaling, Danvers, MA), CHI3L1 (ab77528, 1:1000, Abcam) and GAPDH (MAB374, 1:50000, Merck Millipore, MA, USA). The following day, we incubated the membranes with the secondary fluorescent antibody (C80626-08, Li-Cor Biosciences, Lincoln, Nebraska, USA) for 1 h, in the dark. We detected the signal using the Li-Cor Odyssey® CLx Imaging System (C81205-05, Li-Cor Biosciences). We quantified the density of the bands using ImageJ.

**Tumor secretome-induced pericyte-like differentiation of human mesenchymal stem cells (hMSC).** To address whether the DIPG secretome induced phenotypic changes of hMSC towards B7-H3-expressing pericytes frequently found in the aberrant blood vessels of DIPG, we incubated hMSC-AT with DIPG-conditioned culture medium supernatants for 7 days. We cultured 10^5^ hMSC-AT cells in 3 mL of hMSC culture medium (C-28009, PromoCell) in T25 flasks. The next day, we treated flasks with 3 mL of either hMSC culture medium, pericyte culture medium (P60121, Innoprot, Spain), pHGG medium, DIPG supernatants (DIPG-007, -012 and -021), human astrocytes supernatant, neuroblastoma cell (LAN-1) supernatant, Ewing sarcoma cell (A4573) supernatant, and cytokines dissolved in pHGG medium, including 50 nM human recombinant osteopontin (1433-OP, R&D Systems), or 50 nM human recombinant CHI3L1 (2599-CH-050, R&D Systems). We renewed the culture media every 72 h. After 7 days, we trypsinized and homogenized hMSC-AT cells in RIPA buffer (10 mM Tris-HCl pH 8.0, 1 mM EDTA, 1% Triton X-100, 140mM NaCl; all from Sigma-Aldrich) supplemented with a protease inhibitor cocktail (4693132001, Promega). We determined protein concentrations with the Bradford protein assay. We blocked membranes for 1 h at RT with 5% BSA and incubated them overnight at 4 °C with the primary antibodies for B7-H3 (#14058, 1:1000, Cell Signaling), PD-L1 (ab205921, 1:200, Abcam), NG2 (ab129051, 1:200, Abcam), PDGFRβ (#3169, 1:1000, Cell Signaling) and GAPDH (MAB374, 1:50000, Merck Millipore). The following day, we incubated the membranes with the secondary fluorescent antibody (C80626-08, Li-Cor Biosciences) for 1 h, in the dark. We detected the signal using the Li-Cor Odyssey® CLx Imaging System (C81205-05, Li-Cor Biosciences). We quantified the density of the bands using ImageJ and normalized all values to GAPDH.

**Neutralization of osteopontin.** We neutralized osteopontin in culture media with the neutralizing antibodies 1H3 and 2C5 (ED3002 and ED3001, Kerafast, Boston, MA, USA). To determine the optimal concentration of the anti-osteopontin antibodies we added increasing concentrations (up to 8 µg/mL) to hMSC-AT cells exposed to DIPG-007 supernatants and measured the expression of NG2 and B7-H3 expression after 7 days.

**Tumor secretome-induced changes in brain endothelial cells.** First, we performed a microtubule formation assay of hCMEC/D3 cells exposed to DIPG supernatants. We coated 24-well cell culture plates with 100 µL of unpolymerized geltrex (12760021, Thermo Fisher Scientific) and incubated at 37 °C for 30–45 minutes. We plated 10^5^ hCMEC/D3 cells in 300 µL of either endothelial cell culture medium (C-22011, PromoCell), pHGG medium, DIPG supernatants (DIPG-007, -012 and -021), human astrocytes supernatant, LAN-1 supernatant, A4573 supernatant, and cytokines (50 nM of recombinant osteopontin or CHI3L1) diluted in endothelial cell culture medium. We used endothelial cell culture medium as the reference condition. After 18 h incubation, we took photographs with an inverted photomicroscope at 10x. We analyzed four randomly selected fields from each condition to assess tube formation with the Angiogenesis Analyzer plugin for ImageJ. To determine the complexity of the vascular structures formed in vitro, we measured the total length of the vascular network (in mm), as the addition of the lengths of segments, isolated elements and branches in the analyzed area. We also counted the number of junctions of the new network, adding the total number of vertices formed by the vascular branches.

To address whether osteopontin is involved in protein expression by endothelial cells exposed to DIPG secretomes, we incubated hCMEC/D3 cells for 48 h with DIPG-007 supernatants or 50 nM human recombinant osteopontin, in the presence or absence of osteopontin-neutralizing antibodies 1H3 and 2C5 (2 µg/mL). We then measured the expression of fibronectin (FN1) and BCRP by immunoblotting. We used primary antibodies for FN1 (sc-271098, 1:500, Santa Cruz Biotechnology, Dallas, TX, USA), BCRP (OACD01214, 1:500) and GAPDH (MAB374, 1:50000).

Finally, we studied phenotype changes in co-cultured mesenchymal cells and brain endothelial cells in the presence of DIPG secretomes. We performed a microtubule formation assay of hCMEC/D3 cells co-cultured with hMSC-AT cells exposed to conditioned media. We coated 8-well glass chamber slides with 30 µL of unpolymerized geltrex and incubated at 37 °C for 30–45 minutes. We plated 6x10^4^ hCMEC/D3 and 1.2x10^4^ hMSC-AT cells in 300 µL of either endothelial cell culture medium, mesenchymal medium, pericyte medium, pHGG medium, DIPG supernatants (DIPG-007, -012 and -021), human astrocytes supernatant, LAN-1 supernatant, A4573 supernatant, and cytokines (50 nM of recombinant osteopontin or CHI3L1) dissolved in endothelial cell culture medium. After 18 h incubation, we assessed tube formation.

Cell immunofluorescence. For hMSC-AT cells, we cultured them in 8-well glass chamber slides (PEZGS0816, Merck Millipore). After treatments with conditioned media for 7 days, we fixed the cells with 4% PFA for 30 min, followed by 0.1% Tween 20-PBS permeabilization for 30 min. Then we used 5% BSA as a blocking solution for 1 h. We added primary antibodies for NG2 (ab12905, 1:200, Abcam) and CD90 (ab181469, 1:200, Abcam), kept at 4 ºC overnight.

For cultures of geltrex-embedded human endothelial cells and their co-cultures with hMSC-AT cells, we used 8-well glass chamber slides and treated them with conditioned media for 18 h. Then, we fixed the gels with 80% methanol and 20% DMSO for 30 min at RT. We rehydrated with 50% methanol and 50% PBS for 1 h at RT. Finally, we added 20% methanol and 80% PBS followed by a permeabilization process with 0.1% Tween 20-PBS at RT for 1 h. Then, we added blocking solution (10% fetal bovine serum –FBS-, 5% BSA in PBS) for 2-4 h. We washed with 0.1% Tween 20-Tris Buffered Serum (TBS-T) for 1 h and added the primary antibody diluted in blocking solution at 4 ºC overnight. Antibodies were endothelial marker CD31 (ab32457, 1:200, Abcam), pericyte marker NG2 (ab129051, 1:200, Abcam) and BBB marker BCRP (ab3380, 1:200, Abcam).

For cultures of pHGG tumorspheres and RH cells (used as negative control) we embedded them in optimal cutting temperature compound and posteriorly sliced them into 4-μm sections. First we fixed the cells with 4% PFA for 30 min, followed by 0.1% Tween 20-PBS permeabilization for 30 min. Then we used 5% BSA as a blocking solution for 1 h. We incubated the primary antibodies for osteopontin (ab8448, 1:200, Cell Signaling) and CHI3L1 (ab77528, 1:200, Abcam).

For all the above-mentioned preparations, we used the secondary antibodies anti-mouse-Alexa-488 (A32723, 1:1000, Invitrogen), anti-rabbit-Alexa-594 (A21207, 1:1000, Invitrogen) or anti-rabbit-Alexa-488 (A11034, 1:1000, Invitrogen) for 1 h. Finally, we added DAPI (1:5000, 1 min) and mounted the preparations using Vectashield Antifade Mountant (NC9532821, Fisher Scientific). We obtained images with the Leica Thunder Imager Live cell and 3D Assay software.

**Tumor secretome-induced changes in the macrophage phenotype.** To address whether the DIPG secretome induced polarization of the tumor microglia towards the M2-like phenotype, we exposed macrophage cultures to cell culture-conditioned medium. First, we isolated PBMC from blood buffy coats by density gradient centrifugation gradient separation using Histopaque®-1077 (10771, Sigma-Aldrich). We then cultured cells in macrophage medium (X-VIVO 15, Lonza, Basel, Switzerland) supplemented with 2% human serum, in T75 cell culture flasks for 2 h. To select the adherent macrophages we removed the medium with the non-adherent cells and added fresh macrophage medium supplemented with 2% human serum and 10 ng/mL M-CSF (300-25, Peprotech, Rocky Hill, NJ, USA). We considered macrophage cultures were established after 3 days at 37 ºC in a 5% CO_2_.

We plated 2 x 10^6^ macrophages per well in 6-well plates, in a total of 2 mL. The next day, we exposed them to experimental conditions including an M1 cocktail composed by 20 ng/mL IFN-γ, 20 ng/mL tumor necrosis factor-α (TNF-α, 300-01A) and 100 ng/mL lipopolysaccharide (LPS, L2630, Sigma-Aldrich) to obtain M1-like polarized cells, and an M2 cocktail consisting of 10 ng/mL transforming growth factor-β (TGF-β, 100-21) and 10 ng/mL interleukin 10 (IL-10, 200-10), all from PeproTech. Other experimental conditions included pHGG medium, DIPG supernatant (DIPG-007), and 50 nM recombinant osteopontin dissolved in pHGG medium. We incubated the macrophages for 24 h and then harvested and incubated them with anti-CD11b-Alexa647 (557686, BD Pharmingen, San Diego, CA, USA), anti-CD80-PE (ab69778, Abcam), anti-CD163-FITC (563697, BD Pharmingen) and DAPI for 20 min at 4 ºC in the dark, washed twice and analyzed using a BD FacsCanto™ II Cytometer (BD biosciences, Franklin Lakes, NJ, USA). We selected CD11b^+^ living cells (macrophages) and analyzed them using Infinicyt™ software (Cytognos, Santa Marta de Tormes, Spain). To mitigate the variability in the basal levels of macrophage polarization, we expressed the results as the ratio of CD163^+^ cells (M2-like) to CD80^+^cells (M1-like). In some of these experiments we quantified IL-6 and MMP-9 in the supernatants, using ELISA kits D6050B (R&D Systems) and KE00164 (Proteintech, Rosemond, IL, USA), respectively.

To address the role of osteopontin in the macrophage polarization process, we exposed PBMC-derived macrophages to DIPG secretomes (DIPG-007) in the absence or presence of osteopontin-neutralizing antibodies 1H3 and 2C5 (2 µg/mL). We then addressed the expression of CD163 by flow cytometry and quantified IL-6 and MMP-9 in the supernatants using ELISA.

To address whether DIPG cells modified the migration and invasion of macrophages, we performed a transwell assay using DIPG supernatant and osteopontin as chemoattractants. We plated 2.5 x 10^4^ macrophages per well in 200 μL of macrophage medium in the upper chamber of the transwells (CLS3422-48EA, Merck Millipore), previously coated with geltrex, and FBS-free macrophage medium in the inferior chamber. After 24 h, we filled the lower chamber with either 200 μL of either 10% FBS (12389802, Thermo Fisher Scientific) dissolved in pHGG medium (positive control), 0.1% BSA dissolved in pHGG medium (negative control), or the experimental conditions including DIPG supernatant (DIPG-007), or 50 nM recombinant osteopontin dissolved in pHGG medium. We also included one experimental control of pHGG medium.

After incubation for 72 h at 37 ºC and 5% CO2, we removed the cells on the upper surface of the membrane with a dry cotton swab, fixed the cells attached to the bottom with 4% paraformaldehyde and stained them with 1% crystal violet (548-62-9, Sigma-Aldrich) for 30–60 min. We obtained photographs at 20x using an inverted microscope and counted the cells of randomly selected fields (12 from each condition).
