# Supplementary material for: Proteins of the cancer cell secretome induce the protumoral microenvironment of diffuse intrinsic pontine glioma
Source: Neurooncol Adv. 2025 Jun 19;7(1):vdaf132. doi: 10.1093/noajnl/vdaf132 (PMC12284641; doi:10.1093/noajnl/vdaf132)
Supplement: vdaf132_suppl_Supplementary_Tables_S1-S10 [file vdaf132_suppl_supplementary_tables_s1-s10.docx]

**Table S1.** Patient-derived pHGG models.

| **Primary culture ID** | **Sex** | **Original tumor location** | **Original tumor histology** | **pHGG subclass** | **Histone Mutation** | **Assays performed in this study** |
| --- | --- | --- | --- | --- | --- | --- |
| HSJD-DIPG-007 | M | Pons | pHGG | DMG, H3 K27-altered | *H3F3A* K27M | RT-qPCR, CA, E, IF-Fr |
| HSJD-DIPG-008 | M | Pons | pHGG | DMG, H3 K27-altered | *H3F3A* K27M | RT-qPCR, IF-Fr |
| HSJD-DIPG-011 | F | Pons | pHGG | DMG, H3 K27-altered | *H3F3A* K27M | RT-qPCR |
| HSJD-DIPG-012 | M | Pons | pHGG | DMG, H3 K27-altered | *H3F3A* K27M | RT-qPCR, CA, E, IF-Fr |
| HSJD-DIPG-013 | F | Pons | pHGG | DMG, H3 K27-altered | *H3F3A* K27M | RT-qPCR, CA, E, |
| HSJD-DIPG-014 | F | Pons | pHGG | DMG, H3 K27-altered | *H3F3A* K27M | RT-qPCR, CA, E, |
| HSJD-DIPG-017 | M | Pons | pHGG | DMG, H3 K27-altered | *H3F3A* K27M | CA, E |
| HSJD-DIPG-019 | M | Pons | pHGG | DMG, H3 K27-altered | *H3F3A* K27M | CA, E, IF-Fr |
| HSJD-DIPG-021 | F | Pons | pHGG | DMG, H3 K27-altered | *H3F3A* K27M | CA, E |

Abbreviations: F, female; M, male; CA, cytokine array; E, ELISA; IF-Fr, immunofluorescence in frozen samples; RT-qPCR, real time-quantitative polymerase chain reaction.

**Table S2.** Xenograft models.

| **Model ID** | **Engraftment sites in mice** | **Tumor type** | **Genetic alterations** | **Refs.** |
| --- | --- | --- | --- | --- |
| HSJD-DMG-005 | intracerebral and s.c. | DMG, H3 K27-altered | *H3.3* K27M | [1] |
| HSJD-GBM-001 | intracerebral and s.c. | pHGG, H3-wildtype and IDH-wildtype | *H3* wild type | [1] |
| HSJD-ES-002 | s.c. | Ewing sarcoma | *EWSR1-FLI1* fusion | [2] |
| HSJD-ES-013 | s.c. | Ewing sarcoma | *EWSR1-FLI1* fusion | [3] |
| HSJD-NB-007 | s.c. | Neuroblastoma | *MYCN* amplification | [4] |
| HSJD-WT-5 | s.c. | Wilms tumor | *TP53* mutation | - |
| HSJD-ARMS-7 | s.c. | Alveolar rhabdomyosarcoma | *PAX7-FKHR* fusion | [5] |

Abbreviations: s.c., subcutaneous; Refs., references.

References: [1] Balaguer-Lluna L, Olaciregui NG, Aschero R, et al. Establishment of xenografts and methods to evaluate tumor burden for the three most frequent subclasses of pediatric-type diffuse high grade gliomas. J Neurooncol. 2025; 172(3):599-611. [2] Castillo-Ecija H, Monterrubio C, Pascual-Pasto G, et al. Treatment-driven selection of chemoresistant Ewing sarcoma tumors with limited drug distribution. J Control Release. 2020; 324:440-449. [3] Pascual-Pasto G, Resa-Pares C, Castillo-Ecija H, et al. Low Bcl-2 is a robust biomarker of sensitivity to nab-paclitaxel in Ewing sarcoma. Biochem Pharmacol. 2023; 208:115408. [4] Aschero R, Castillo-Ecija H, Baulenas-Farres M, et al. Prognostic value of xenograft engraftment in patients with metastatic high-risk neuroblastoma. Pediatric blood & cancer. 2023; 70(6):e30318. [5] Castillo-Ecija H, Pascual-Pasto G, Perez-Jaume S, et al. Prognostic value of patient-derived xenograft engraftment in pediatric sarcomas. The journal of pathology. Clinical research. 2021; 7(4):338-349.

**Table S3.** TaqMan probes references for the RT-qPCR assays.

| **Gene symbol** | **Protein** | **TaqMan probe** |
| --- | --- | --- |
| *CD274* | PD-L1 | Hs01125301_m1 |
| *CD273* | PD-L2 | Hs01057777_m1 |
| *CD152* | CTLA-4 | Hs03044418_m1 |
| *CD276* | B7-H3 | Hs00987207_m1 |
| *IDO1* | Idoleamine 2,3-dioxygenase 1 | Hs00984148_m1 |
| *IDO2* | Idoleamine 2,3-dioxygenase 1 | Hs01589373_m1 |
| *TDO* | Tryptophan 2,3-dioxygenase | Hs00194611_m1 |
| *PTGES* | Prostaglandin E synthase | Hs00610420_m1 |
| *PTGS2* | Prostaglandin-endoperoxide synthase 2 | Hs00153133_m1 |
| *HLA-A* | Major histocompatibility complex, class I, A | Hs010588061_g1 |
| *HLA-B* | Major histocompatibility complex, class I, B | Hs00818803_g1 |
| *HLA-C* | Major histocompatibility complex, class I, C | Hs03044135_m1 |
| *HLA-DRA* | Major histocompatibility complex, class II, DR alpha | Hs00219575_m1 |
| *HLA-DRB1* | Major histocompatibility complex, class II, DR beta 1 | Hs99999917_m1 |
| *B2M* | Beta-2-microglobulin | Hs00984230_m1 |
| *CIITA* | Class II major histocompatibility complex transactivator | Hs00172094_m1 |
| *HSPA1A* | Heat shock protein family A (Hsp70) member 1A | Hs00359163_s1 |
| *HSPA4* | Heat shock protein family A (Hsp70) member 4 | Hs00382884_m1 |
| *HSPD1* | Heat shock protein family A (Hsp60) member 1 | Hs01036753_g1 |
| *HSP90AA1* | Heat shock protein 90 alpha family class A member 1 | Hs00743767_sH |
| *TGFB1* | Transforming growth factor beta 1 | Hs00998133_m1 |
| *TGFB2* | Transforming growth factor beta 2 | Hs00234244_m1 |
| *TGFB3* | Transforming growth factor beta 3 | Hs01086000_m1 |
| *STAT3* | Signal transducer and activator of transcription 3 | Hs00374280_m1 |
| *CALR* | Calreticulin | Hs00189032_m1 |
| *HMGB1* | High mobility group box 1 | Hs01923466_g1 |
| *HPRT1* | Hypoxanthine phosphoribosyltansferase 1 | Hs02800695_m1 |
| *SPP1* | Osteopontin | Hs00959010_m1 |
| *CHI3L1* | Chitinase 3-like 1 | Hs01072228_m1 |
| *TBP* | TATA-box binding protein | Hs00427620_m1 |

**Table S4.** Human brainstem samples used for the immunohistochemistry analyses.

| **Patient code^1^** | **Sample type** | **Sex** | **Disease stage at sampling** | **Prior treatment^2^** | **Tumor histology** | **Diagnosis** | **Histone mutation** | **Histone immunostaining^3^** |
| --- | --- | --- | --- | --- | --- | --- | --- | --- |
| HSJD-CNS-001 | Nontumor control necropsy | M | NA | Unknown | NA | Cardiac arrest | Not studied | Not studied |
| HSJD-CNS-002 | Nontumor control necropsy | M | NA | Unknown | NA | Steinert syndrome | Not studied | Not studied |
| HSJD-CNS-003 | Nontumor control necropsy | M | NA | Unknown | NA | Fetal death | Not studied | Not studied |
| HSJD-CNS-004 | Nontumor control necropsy | F | NA | Unknown | NA | Tetralogy of Fallot | Not studied | Not studied |
| HSJD-CNS-005 | Nontumor control necropsy | F | NA | Unknown | NA | Unknown | Not studied | Not studied |
| HSJD-DIPG-021 | Tumor biopsy | F | Diagnosis | Corticosteroids only | pHGG | DMG, H3 K27-altered | Not studied | K27-altered |
| HSJD-DIPG-023 | Tumor biopsy | F | Diagnosis | Corticosteroids only | pHGG | DMG, H3 K27-altered | Not studied | K27-altered |
| HSJD-DIPG-018 | Tumor biopsy | F | Diagnosis | Corticosteroids only | pHGG | DMG, H3 K27-altered | *HIST1H3B* K27M | K27-altered |
| HSJD-DIPG-019 | Tumor biopsy | M | Diagnosis | Corticosteroids only | pHGG | DMG, H3 K27-altered | *H3F3A* K27M | K27-altered |
| HSJD-DIPG-022 | Tumor biopsy | M | Progression | RT, vinorelbine, methotrexate, etoposide, rapamycin, irinotecan, temozolomide, bevacizumab | pHGG | H3-wildtype and IDH-wildtype pHGG | Wild type | No alteration |
| HSJD-DIPG-038 | Tumor biopsy | F | Diagnosis | Corticosteroids only | pHGG | DMG, H3 K27-altered | Not studied | K27-altered |
| HSJD-DIPG-040 | Tumor biopsy | F | Diagnosis | Corticosteroids only | pHGG | DMG, H3 K27-altered | Not studied | K27-altered |
| HSJD-DIPG-002 | Tumor necropsy | F | Progression | RT, irinotecan-cisplatin | pHGG | DMG, H3 K27-altered | *H3F3A* K27M | K27-altered |
| HSJD-DIPG-018 | Tumor necropsy | F | Progression | RT, bevacizumab | pHGG | DMG, H3 K27-altered | *HIST1H3B* K27M | K27-altered |
| HSJD-DIPG-004 | Tumor necropsy | F | Progression | RT, irinotecan-cisplatin, nimotuzumab | pHGG | DMG, H3 K27-altered | *HIST1H3B* K27M | K27-altered |
| HSJD-DIPG-014 | Tumor necropsy | F | Progression | RT, rapamycin, irinotecan | pHGG | DMG, H3 K27-altered | *H3F3A* K27M | K27-altered |
| HSJD-DIPG-015 | Tumor necropsy | M | Progression | RT, temozolomide, etoposide | pHGG | DMG, H3 K27-altered | *H3F3A* K27M | K27-altered |
| HSJD-DIPG-001 | Tumor necropsy | F | Progression | RT, temozolomide | pHGG | DMG, H3 K27-altered | *H3F3A* K27M | K27-altered |
| HSJD-DIPG-008 | Tumor necropsy | M | Progression | RT, irinotecan-cisplatin, nimotuzumab- vinorelbine, bevacizumab | pHGG | DMG, H3 K27-altered | *H3F3A* K27M | K27-altered |
| HSJD-DIPG-003 | Tumor necropsy | M | Progression | RT, irinotecan-cisplatin, temozolomide, nimotuzumab, bevacizumab | pHGG | DMG, H3 K27-altered | *H3F3A* K27M | K27-altered |
| HSJD-DIPG-010 | Tumor necropsy | F | Progression | RT, nimotuzumab-vinorelbine | pHGG | DMG, H3 K27-altered | *H3F3A* K27M | K27-altered |
| HSJD-DIPG-022 | Tumor necropsy | M | Progression | RT, vinorelbine, methotrexate, etoposide, rapamycin, irinotecan, temozolomide, bevacizumab | pHGG | H3-wildtype and IDH-wildtype pHGG | Wild type | No alteration |
| HSJD-DIPG-016 | Tumor necropsy | F | Progression | RT, temozolomide | pHGG | DMG, H3K27-altered | *H3F3A* K27M | K27-altered |
| HSJD-DIPG-005 | Tumor necropsy | F | Progression | RT, irinotecan-cisplatin, temozolomide, nimotuzumab, bevacizumab | pHGG | DMG, H3K27-altered | Not studied | K27-altered |
| HSJD-DIPG-009 | Tumor necropsy | F | Progression | RT, nimotuzumab-vinorelbine | pHGG | DMG, H3K27-altered | Not studied | K27-altered |
| HSJD-DIPG-039 | Tumor necropsy | M | Progression | RT, temozolomide | pHGG | DIPG | Not studied | Not studied |
| HSJD-DIPG-007 | Tumor necropsy | M | Progression | RT, irinotecan-cisplatin | pHGG | DMG, H3 K27-altered | *H3F3A* K27M | K27-altered |
| HSJD-DIPG-036 | Tumor necropsy | F | Progression | RT, immunotherapy | pHGG | DMG, H3 K27-altered | *H3F3A* K27M | K27-altered |
| HSJD-DIPG-041 | Tumor necropsy | M | Progression | Unknown | pHGG | DIPG | Not studied | Not studied |
| HSJD-DIPG-006 | Tumor necropsy | F | Progression | RT, irinotecan-cisplatin, temozolomide, nimotuzumab, bevacizumab | pHGG | DMG, H3K27-altered | Not studied | K27-altered |

^1^Note there are paired biopsy-necropsy samples included for patients coded HSJD-DIPG-018 ad HSJD-DIPG-022.

^2^All patients with DIPG received corticosteroids.

^3^Immunostaining assays for tri-methyl-histone H3 (Lys27) and/or histone H3.3 K27M. The K27-altered phenotype consisted in negative staining for tri-methyl-histone H3 and positive staining for histone H3.3 K27M.

Abbreviations: F, female; M, male; NA, not applicable.

**Table S5.** Human brainstem samples used for the RT-qPCR analysis of genes *CD276* (B7-H3), *CD274* (PD-L1), *CD273* (PD-L2) and *CD152* (CTLA-4).

| **Sample type** | **Patient code** | **Sex** | **Tumor histology** | **Diagnosis** |
| --- | --- | --- | --- | --- |
| Nontumor control necropsy | HSJD-CNS-001 | M | NA | Unknown |
| Nontumor control necropsy | 1701 | M | NA | Unknown |
| Nontumor control necropsy | HSJD-CNS-003 | M | NA | Fetal death |
| Nontumor control necropsy | HSJD-CNS-004 | F | NA | Tetralogy of Fallot |
| Tumor necropsy | HSJD-DIPG-002 | F | pHGG | DMG, H3 K27-altered |
| Tumor necropsy | HSJD-DIPG-018 | F | pHGG | DMG, H3 K27-altered |
| Tumor necropsy | HSJD-DIPG-004 | F | pHGG | DMG, H3 K27-altered |
| Tumor necropsy | HSJD-DIPG-014 | F | pHGG | DMG, H3 K27-altered |
| Tumor necropsy | HSJD-DIPG-015 | M | pHGG | DMG, H3 K27-altered |
| Tumor necropsy | HSJD-DIPG-001 | F | pHGG | DMG, H3 K27-altered |
| Tumor necropsy | HSJD-DIPG-008 | M | pHGG | DMG, H3 K27-altered |
| Tumor necropsy | HSJD-DIPG-003 | M | pHGG | DMG, H3 K27-altered |
| Tumor necropsy | HSJD-DIPG-010 | F | pHGG | DMG, H3 K27-altered |
| Tumor necropsy | HSJD-DIPG-022 | M | pHGG | H3-wildtype and IDH-wildtype pHGG |
| Tumor necropsy | HSJD-DIPG-009 | F | pHGG | DMG, H3K27-altered |
| Tumor necropsy | HSJD-DIPG-036 | F | pHGG | DMG, H3 K27-altered |

Abbreviations: F, female; M, male; NA, not applicable.

**Table S6.** Human brainstem samples used in the proteome array analysis.

| **Sample type** | **Patient code** | **Sex** | **Tumor histology** | **Diagnosis** |
| --- | --- | --- | --- | --- |
| Nontumor control necropsy | HSJD-CNS-001 | M | NA | Cardiac arrest |
| Nontumor control necropsy | HSJD-CNS-002 | M | NA | Steinert syndrome |
| Nontumor control necropsy | HSJD-CNS-003 | M | NA | Fetal death |
| Nontumor control necropsy | HSJD-CNS-004 | F | NA | Tetralogy of Fallot |
| Tumor necropsy | HSJD-DIPG-002 | F | pHGG | DMG, H3 K27-altered |
| Tumor necropsy | HSJD-DIPG-018 | F | pHGG | DMG, H3 K27-altered |
| Tumor necropsy | HSJD-DIPG-004 | F | pHGG | DMG, H3 K27-altered |
| Tumor necropsy | HSJD-DIPG-014 | F | pHGG | DMG, H3 K27-altered |
| Tumor necropsy | HSJD-DIPG-015 | M | pHGG | DMG, H3 K27-altered |
| Tumor necropsy | HSJD-DIPG-036 | F | pHGG | DMG, H3 K27-altered |
| Tumor necropsy | HSJD-DIPG-001 | F | pHGG | DMG, H3 K27-altered |
| Tumor necropsy | HSJD-DIPG-008 | M | pHGG | DMG, H3 K27-altered |
| Tumor necropsy | HSJD-DIPG-003 | M | pHGG | DMG, H3 K27-altered |
| Tumor necropsy | HSJD-DIPG-010 | F | pHGG | DMG, H3 K27-altered |
| Tumor necropsy | HSJD-DIPG-022 | M | pHGG | H3-wildtype and IDH-wildtype pHGG |
| Tumor necropsy | HSJD-DIPG-037 | F | pHGG | DMG, H3 K27-altered |
| Tumor necropsy | HSJD-DIPG-023 | F | pHGG | DMG, H3 K27-altered |
| Tumor necropsy | HSJD-DIPG-021 | F | pHGG | DMG, H3 K27-altered |

Abbreviations: F, female; M, male; NA, not applicable.

**Table S7.** Human brainstem samples analyzed by ELISA.

| **Sample type** | **Patient code** | **Sex** | **Tumor histology** | **Diagnosis** |
| --- | --- | --- | --- | --- |
| Nontumor control necropsy | HSJD-CNS-001 | M | NA | Cardiac arrest |
| Nontumor control necropsy | HSJD-CNS-002 | M | NA | Steinert syndrome |
| Nontumor control necropsy | HSJD-CNS-003 | M | NA | Fetal death |
| Nontumor control necropsy | HSJD-CNS-004 | F | NA | Tetralogy of Fallot |
| Tumor necropsy | HSJD-DIPG-002 | F | pHGG | DMG, H3 K27-altered |
| Tumor necropsy | HSJD-DIPG-018 | F | pHGG | DMG, H3 K27-altered |
| Tumor necropsy | HSJD-DIPG-014 | F | pHGG | DMG, H3 K27-altered |
| Tumor necropsy | HSJD-DIPG-015 | M | pHGG | DMG, H3 K27-altered |
| Tumor necropsy | HSJD-DIPG-036 | F | pHGG | DMG, H3 K27-altered |
| Tumor necropsy | HSJD-DIPG-001 | F | pHGG | DMG, H3 K27-altered |
| Tumor necropsy | HSJD-DIPG-008 | M | pHGG | DMG, H3 K27-altered |
| Tumor necropsy | HSJD-DIPG-003 | M | pHGG | DMG, H3 K27-altered |
| Tumor necropsy | HSJD-DIPG-022 | M | pHGG | H3-wildtype and IDH-wildtype pHGG |

Abbreviations: F, female; M, male; NA, not applicable.

**Table S8.** Cerebrospinal fluid samples analyzed by ELISA.

| **Sample** | **Diagnosis** |
| --- | --- |
| LCR01 | Unknown |
| LCR04 | Unknown |
| LCR06 | Unknown |
| LCR10 | Unknown |
| CTRL019 | Unknown |
| CTRL021 | Unknown |
| CTRL023 | Unknown |
| CTRL024 | Unknown |
| CTRL025 | Unknown |
| CTRL026 | Unknown |
| CTRL027 | Unknown |
| CTRL028 | Unknown |
| CTRL111 | Hydrocephalus |
| CTRL113 | Hydrocephalus |
| CTRL114 | Cranioplasty |
| CTRL116 | Papilledema |
| CTRL125 | Hydrocephalus |
| CTRL0023 | Hydrocephalus |
| HSJD-DIPG-037 | DMG, H3 K27-altered |
| HSJD-DIPG-021 | DMG, H3 K27-altered |
| HSJD-DIPG-023 | DMG, H3 K27-altered |
| DIPG-DC-004 | DIPG |
| DIPG-DC-006 | DIPG |
| DIPG-DC-007 | DIPG |
| HSJD-DIPG-038 | DMG, H3 K27-altered |
| DIPG-DC-009 | DIPG |
| DIPG-DC-010 | DIPG |
| DIPG-DC-011 | DIPG |
| HSJD-DIPG-036 | DMG, H3 K27-altered |
| HSJD-DIPG-018 | DMG, H3 K27-altered |
| HSJD-DIPG-022 | H3-wildtype and IDH-wildtype pHGG |
| HSJD-DIPG-034 | DMG, H3 K27-altered |
| HSJD-GBM-026 | DMG, H3 K27-altered |
| HSJD-DIPG-024 | DMG, H3 K27-altered |
| DIPG-016 | DIPG |
| DIPG-017 | DMG, H3 K27-altered |

**Table S9.** Human brainstem samples analyzed by immunoblotting.

| **Sample type** | **Patient code** | **Sex** | **Tumor histology** | **Diagnosis** |
| --- | --- | --- | --- | --- |
| Nontumor control necropsy | HSJD-CNS-001 | M | NA | Cardiac arrest |
| Nontumor control necropsy | HSJD-CNS-002 | M | NA | Steinert syndrome |
| Nontumor control necropsy | HSJD-CNS-003 | M | NA | Fetal death |
| Tumor necropsy | HSJD-DIPG-002 | F | pHGG | DMG, H3 K27-altered |
| Tumor necropsy | HSJD-DIPG-018 | F | pHGG | DMG, H3 K27-altered |
| Tumor necropsy | HSJD-DIPG-004 | F | pHGG | DMG, H3 K27-altered |
| Tumor necropsy | HSJD-DIPG-014 | F | pHGG | DMG, H3 K27-altered |
| Tumor necropsy | HSJD-DIPG-015 | M | pHGG | DMG, H3 K27-altered |
| Tumor necropsy | HSJD-DIPG-036 | F | pHGG | DMG, H3 K27-altered |
| Tumor necropsy | HSJD-DIPG-008 | M | pHGG | DMG, H3 K27-altered |
| Tumor necropsy | HSJD-DIPG-003 | M | pHGG | DMG, H3 K27-altered |
| Tumor necropsy | HSJD-DIPG-022 | M | pHGG | H3-wildtype and IDH-wildtype pHGG |

Abbreviations: F, female; M, male; NA, not applicable.

**Table S10.** Human brainstem samples used in the immunofluorescence assay of NG2 and CD90.

| **Sample type** | **Patient code** | **Sex** | **Tumor histology** | **Diagnosis** |
| --- | --- | --- | --- | --- |
| Nontumor control necropsy | HSJD-CNS-002 | M | - | Steinert syndrome |
| Nontumor control necropsy | HSJD-CNS-003 | M | - | Fetal death |
| Tumor necropsy | HSJD-DIPG-018 | F | pHGG | DMG, H3 K27-altered |
| Tumor necropsy | HSJD-DIPG-036 | F | pHGG | DMG, H3 K27-altered |
| Tumor necropsy | HSJD-DIPG-003 | M | pHGG | DMG, H3 K27-altered |
| Tumor necropsy | HSJD-DIPG-005 | F | pHGG | DMG, H3 K27-altered |

Abbreviations: F, female; M, male; NA, not applicable.
