# Supplementary material for: Proteins of the cancer cell secretome induce the protumoral microenvironment of diffuse intrinsic pontine glioma
Source: Neurooncol Adv. 2025 Jun 19;7(1):vdaf132. doi: 10.1093/noajnl/vdaf132 (PMC12284641; doi:10.1093/noajnl/vdaf132)
Supplement: vdaf132_suppl_Supplementary_Figures_S1-S7 [file vdaf132_suppl_supplementary_figures_s1-s7.docx]

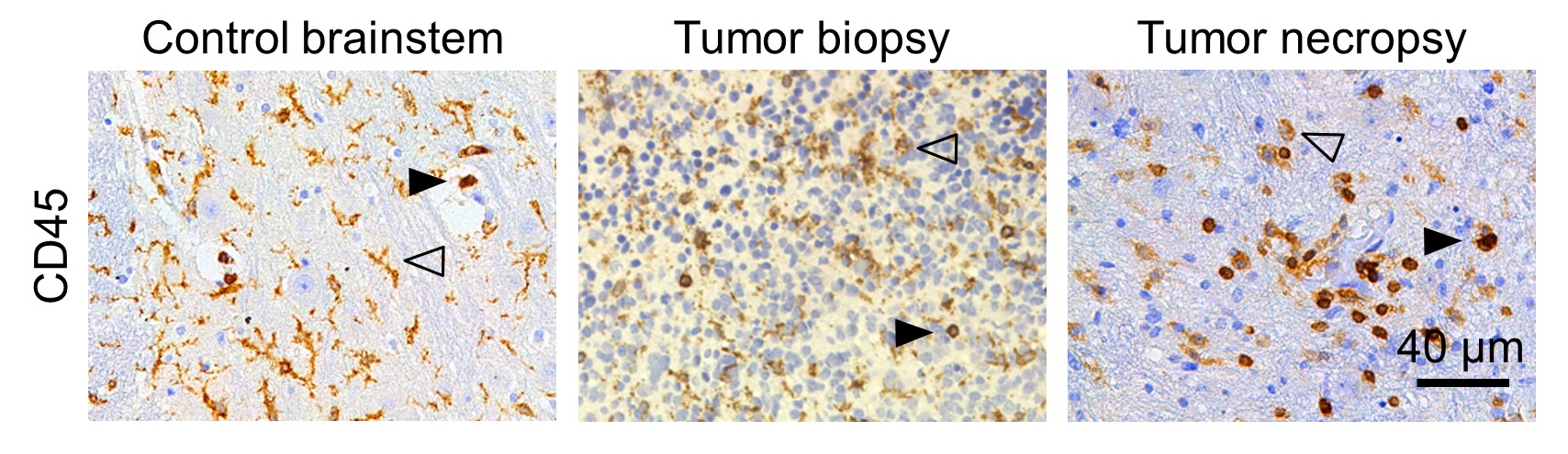


**Figure S1.** **Staining of CD45 in representative samples of the study (control brainstem, tumor biopsy and tumor necropsy).** Black arrows indicate CD45^high^ staining, corresponding to round lymphocytes. Empty arrows indicate CD45^low^ staining, corresponding to ramified microglia/macrophages.


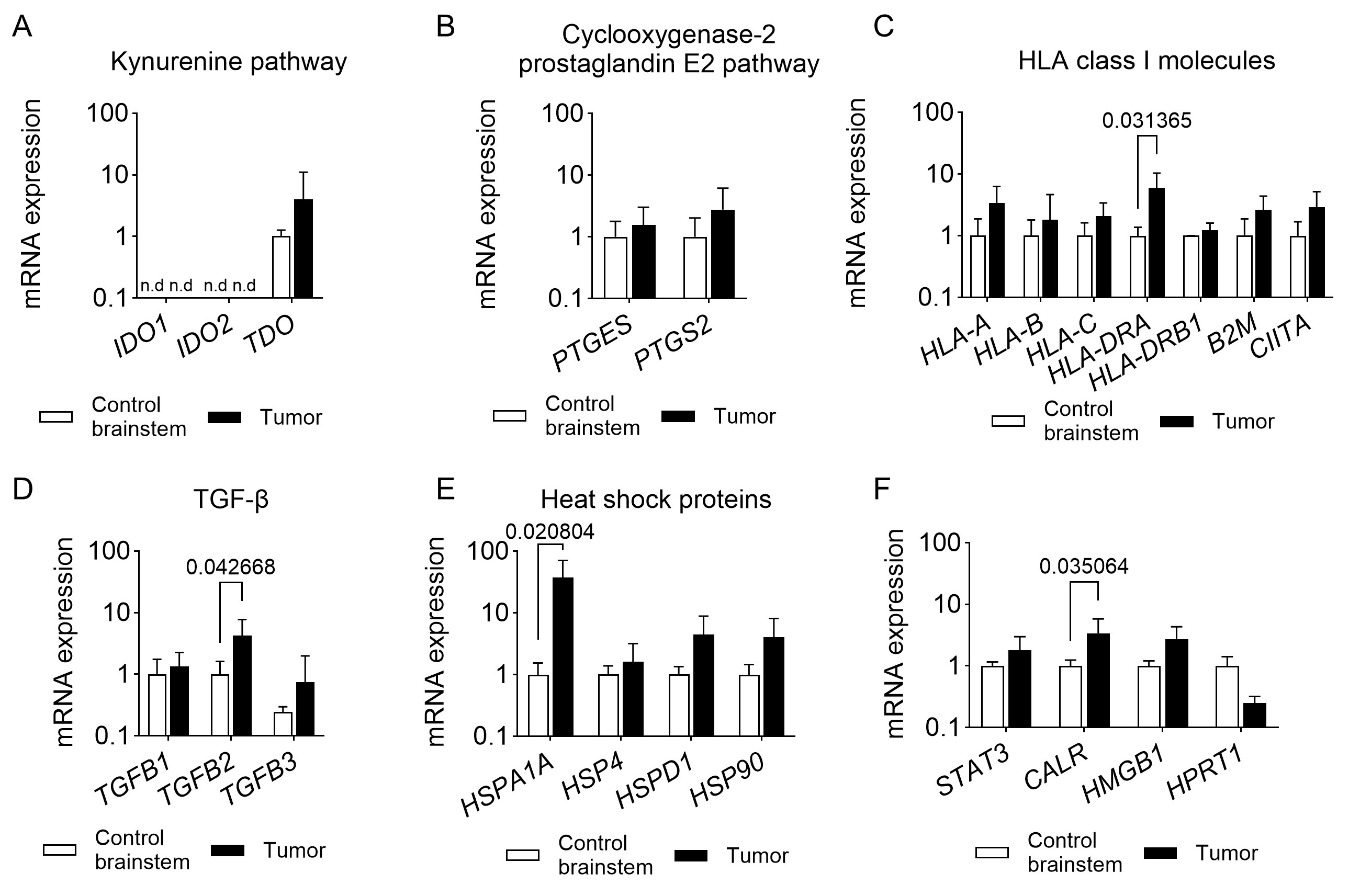


**Figure S2.** **Expression of selected genes related to immune escape mechanisms in DIPG**. (A) Expression of genes associated to kynurenine pathway. (B) Expression of genes associated to cyclooxygenase-2 prostaglandin E2 pathway. (C) Expression of the gene constant region (B2M), the polymorphic regions (HLA-A/B/C) of the HLA class I and HLA class II molecules (HLA-DRA/DRB1) and the MHC-II transactivator (CIITA). (D) Expression of TGF-β isoforms. (E) Expression of heat shock proteins. (F) Expression of other immunomodulatory molecules associated with glioma progression. Bars and error bars represent means and SD of healthy brainstem (n = 3) or tumor samples (n = 11). Statistics: unpaired t test with Holm-Šídák's multiple comparisons. Abbreviations: n.d: not detected.


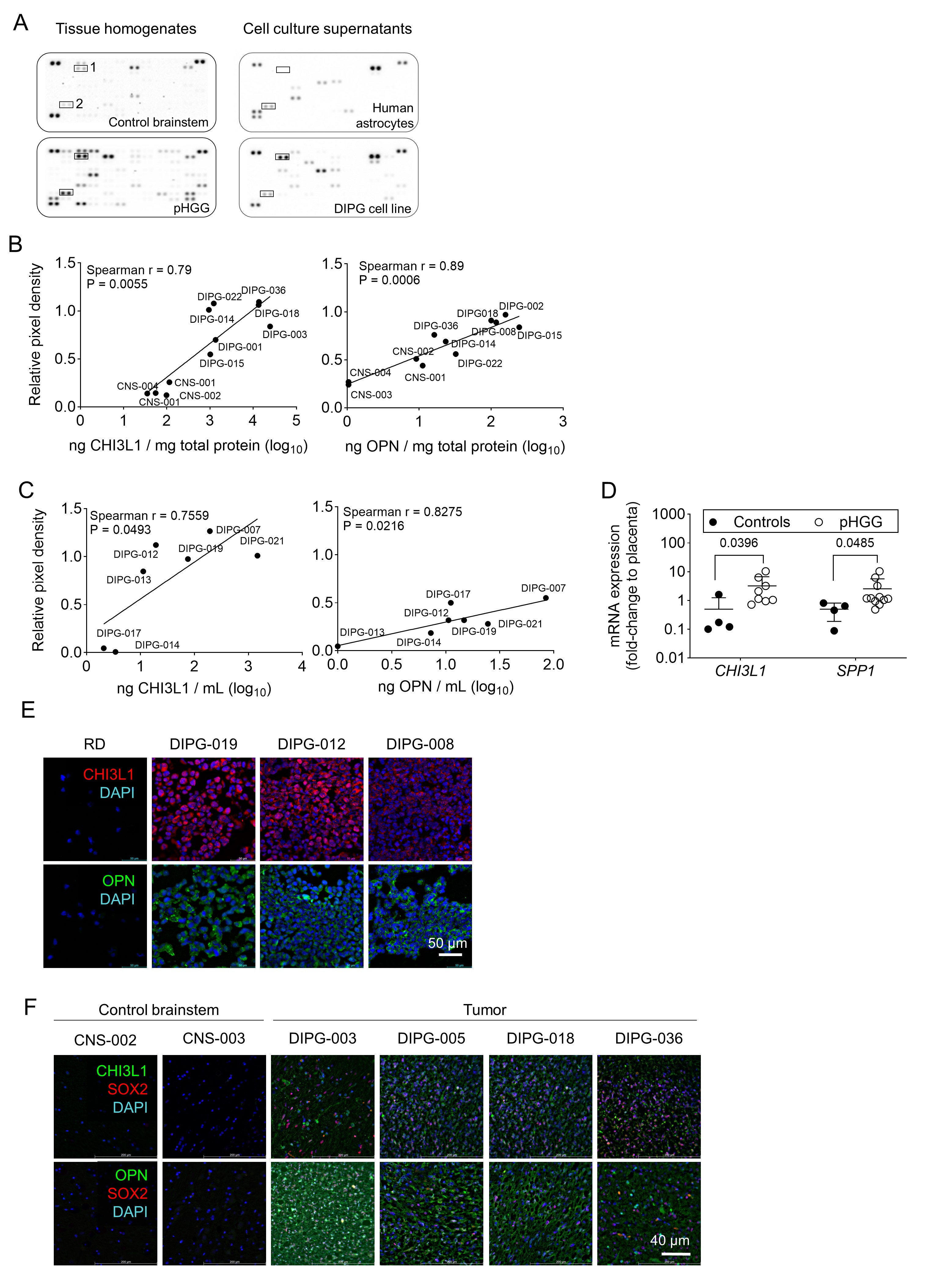


**Figure S3. Analysis of cytokines in pHGG and control samples.** (A) Representative images of array membranes corresponding to homogenized tissue samples from control brainstem (CNS-003) and pHGG (DIPG-018) and cell culture supernatants from human astrocytes and a DIPG cell line (DIPG-012). The numbered pairs of spots correspond to CHI3L1 (1) and osteopontin (2). (B) Correlation of the intensity of the spots of the cytokine arrays (Y axis) and the ELISA results (X axis) in tissue lysates (n = 11). The line indicates the simple regression line. Individual data with sample identifications are represented. (C) Correlation of the the intensity of the spots of cytokine arrays and the ELISA results in DIPG cell supernatants (n = 7). The line indicates the simple regression line. Individual data with sample identifications are represented. (D) mRNA expression of *CHI3L1* and *SPP1* in control brainstem (controls; n = 4) and tumor tissue samples (pHGG; n = 10). Results are relativized to the expression in placenta. (E) Immunofluorescence of osteopontin and CHI3L1 in a cell line without expression (RD; rhabdomyosarcoma) and DIPG tumorspheres. (F) Immunofluorescence of CHI3L1 and SOX2, or OPN and SOX2, in control brainstem (n = 2) and tumor tissue samples (n = 4). Note that areas merging green and red appear as pink in the images.


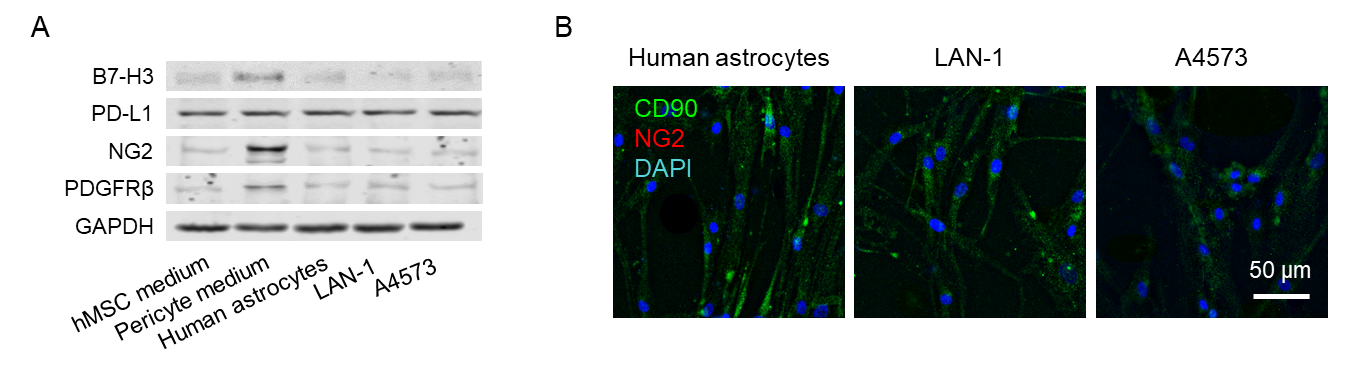


**Figure S4. Pericyte markers in hMSC exposed to cell culture-conditioned media.** (A) Immunoblotting of B7-H3, PD-L1, NG2 and PDGFR-β in hMSC-AT exposed for 7 days to fresh culture media and cell culture-conditioned media (human astrocytes, LAN-1 and A4573 cells). (B) Immunofluorescence of CD90 and NG2 in hMSC-AT incubated with conditioned media for 7 days.


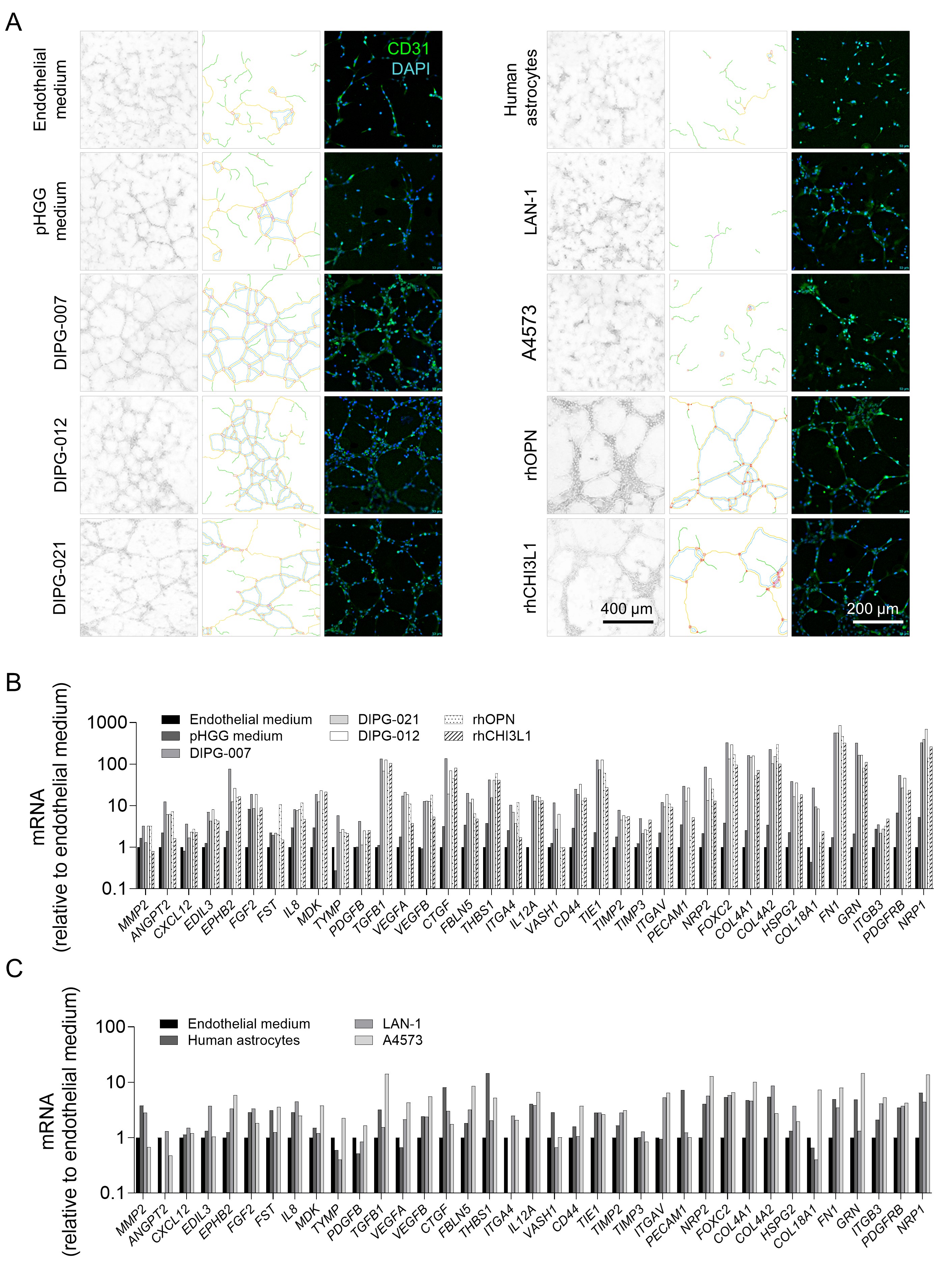


**Figure S5. Angiogenic events in hCMEC/D3 cells exposed to cell culture-conditioned media.** (A) Representative images of the tube formation assay of hCMEC/D3 cells exposed to fresh culture media, cell culture-conditioned media (DIPG cell lines, human astrocytes, LAN-1 and A4573), and recombinant osteopontin and CHI3L1 (rhOPN or rhCHI3L1) for 18 h. From left to right, the three columns of images are the bright field, the software-generated diagram of the network (with master junctions in red, branches in green, the mesh in blue, and master segments in yellow), and the immunofluorescence of CD31. (B) Expression of angiogenesis-related genes included in the TaqMan™ Human Angiogenesis Array in hCMEC/D3 cells exposed to fresh culture media, DIPG-conditioned media, rhOPN, or rhCHI3L1, for 48 h. Values are normalized to those obtained for hCMEC/D3 in endothelial medium. (C) Expression of angiogenic-related genes included in the TaqMan™ Human Angiogenesis Array in hCMEC/D3 cells exposed to fresh culture media or cell culture-conditioned media (human astrocytes, LAN-1 and A4573) for 48 h.


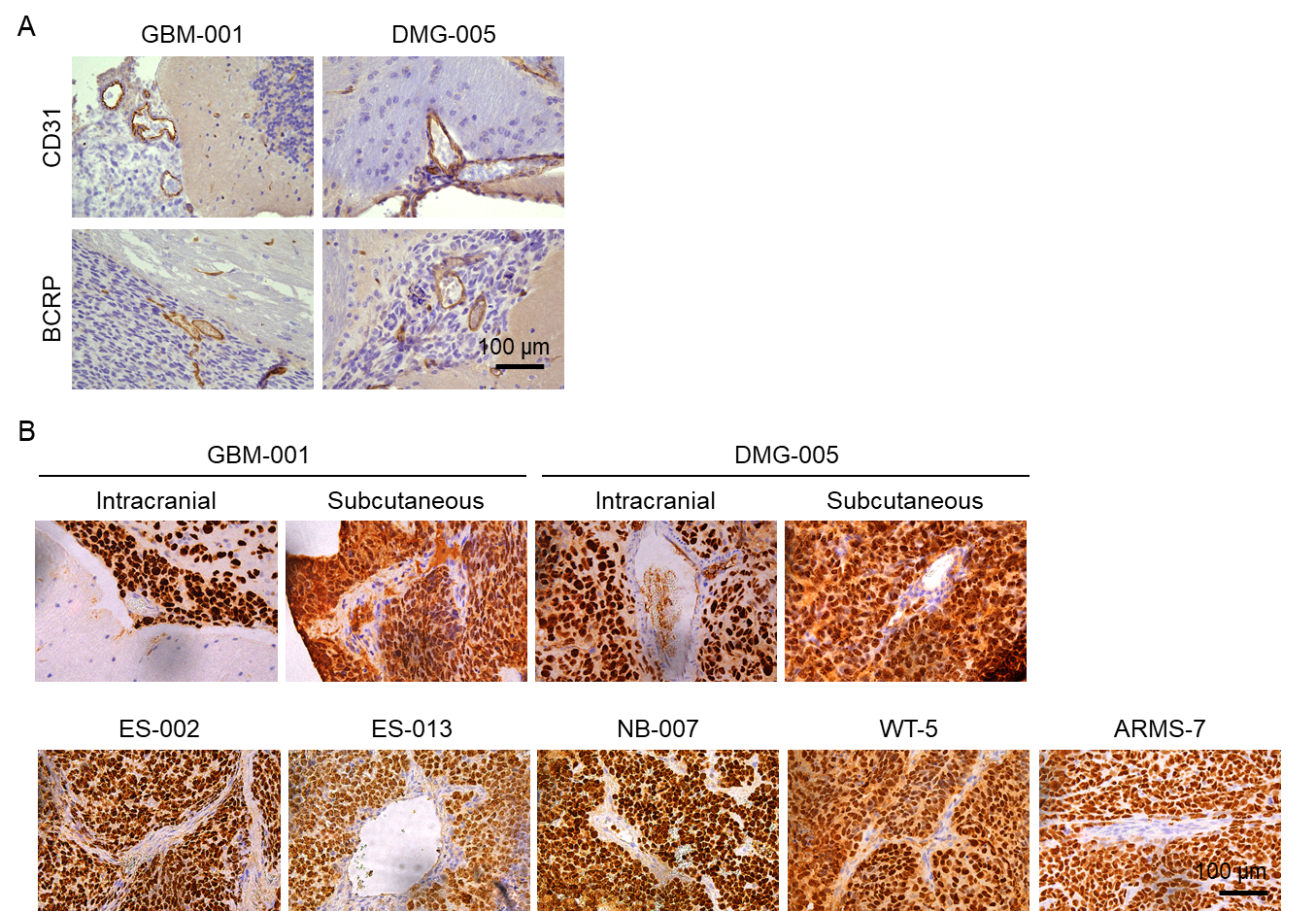


**Figure S6.** **Vasculature markers in human cancer xenografts in mice.** (A) Immunostaining of CD31 and BCRP in the intracranial (orthotopic) xenografts of the pHGG models GBM-001 and DMG-005. (B) Immunostaining of human nuclei (in brown) in intracranial and s.c. xenografts of pHGG, and s.c. xenografts of Ewing sarcoma, neuroblastoma, Wilms tumor and alveolar rhabdomyosarcoma. Note that the nuclei of endothelial cells are not stained in brown, due to their mouse (host) origin.


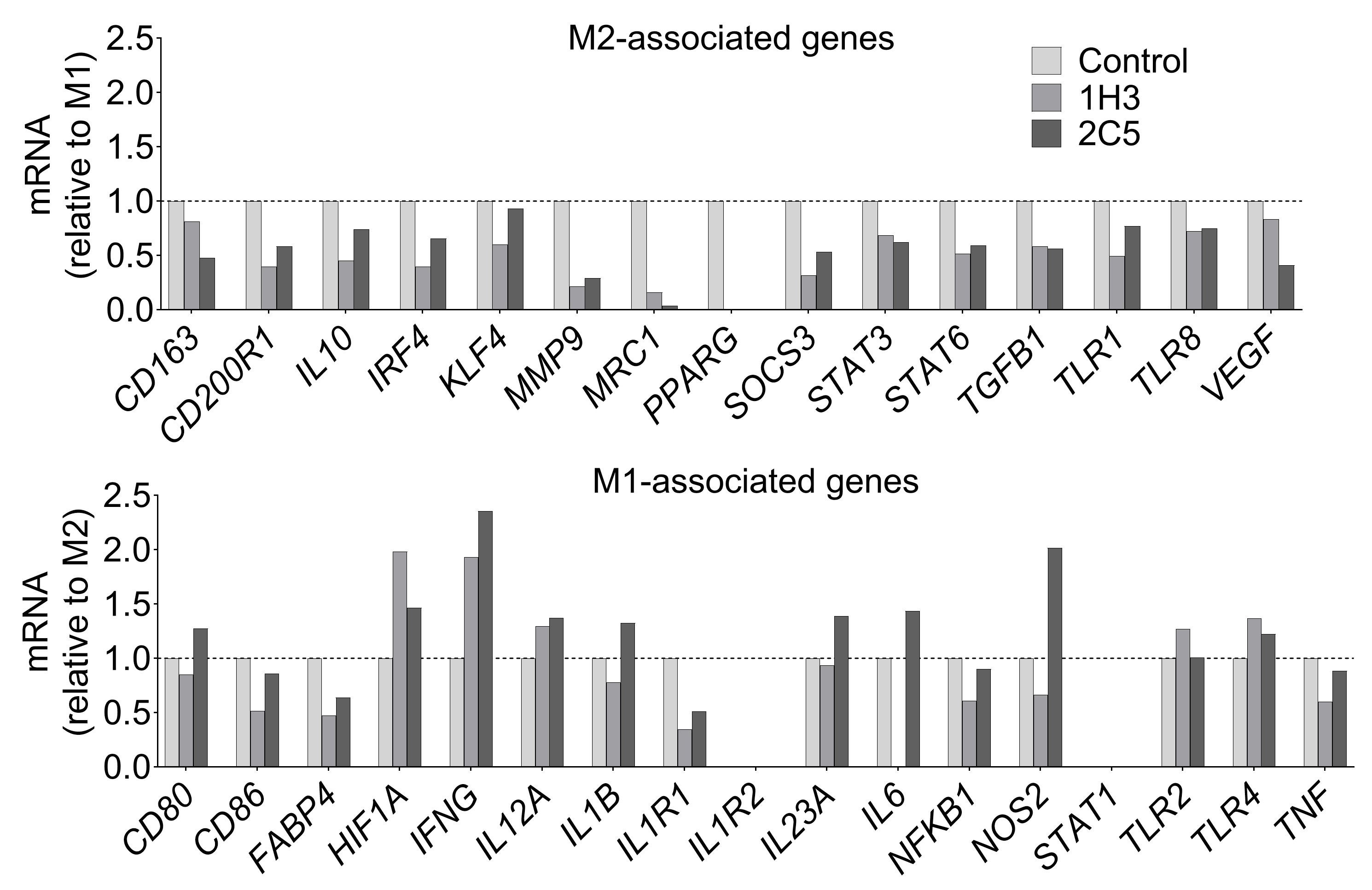


**Figure S7.** **Effect of osteopontin neutralization on the polarization of PBMC-derived macrophages.** Expression of genes included in the GeneQuery™ Human Macrophage Polarization Markers qPCR Array Kit, in PBMC-derived macrophages exposed to DIPG-conditioned medium (DIPG-007, control), or DIPG-conditioned medium supplemented with the osteopontin neutralizing antibodies 1H3 and 2C5 at 2 μg/mL, for 24 h. Values of M1 and M2-associated genes are normalized to those of the control.
